# Cohort Profile: Pregnancy And Childhood Epigenetics (PACE) Consortium

**DOI:** 10.1093/ije/dyx190

**Published:** 2017-09-13

**Authors:** Janine F Felix, Bonnie R Joubert, Andrea A Baccarelli, Gemma C Sharp, Catarina Almqvist, Isabella Annesi-Maesano, Hasan Arshad, Nour Baïz, Marian J Bakermans-Kranenburg, Kelly M Bakulski, Elisabeth B Binder, Luigi Bouchard, Carrie V Breton, Bert Brunekreef, Kelly J Brunst, Esteban G Burchard, Mariona Bustamante, Leda Chatzi, Monica Cheng Munthe-Kaas, Eva Corpeleijn, Darina Czamara, Dana Dabelea, George Davey Smith, Patrick De Boever, Liesbeth Duijts, Terence Dwyer, Celeste Eng, Brenda Eskenazi, Todd M Everson, Fahimeh Falahi, M Daniele Fallin, Sara Farchi, Mariana F Fernandez, Lu Gao, Tom R Gaunt, Akram Ghantous, Matthew W Gillman, Semira Gonseth, Veit Grote, Olena Gruzieva, Siri E Håberg, Zdenko Herceg, Marie-France Hivert, Nina Holland, John W Holloway, Cathrine Hoyo, Donglei Hu, Rae-Chi Huang, Karen Huen, Marjo-Riitta Järvelin, Dereje D Jima, Allan C Just, Margaret R Karagas, Robert Karlsson, Wilfried Karmaus, Katerina J Kechris, Juha Kere, Manolis Kogevinas, Berthold Koletzko, Gerard H Koppelman, Leanne K Küpers, Christine Ladd-Acosta, Jari Lahti, Nathalie Lambrechts, Sabine AS Langie, Rolv T Lie, Andrew H Liu, Maria C Magnus, Per Magnus, Rachel L Maguire, Carmen J Marsit, Wendy McArdle, Erik Melén, Phillip Melton, Susan K Murphy, Tim S Nawrot, Lorenza Nisticò, Ellen A Nohr, Björn Nordlund, Wenche Nystad, Sam S Oh, Emily Oken, Christian M Page, Patrice Perron, Göran Pershagen, Costanza Pizzi, Michelle Plusquin, Katri Raikkonen, Sarah E Reese, Eva Reischl, Lorenzo Richiardi, Susan Ring, Ritu P Roy, Peter Rzehak, Greet Schoeters, David A Schwartz, Sylvain Sebert, Harold Snieder, Thorkild IA Sørensen, Anne P Starling, Jordi Sunyer, Jack A Taylor, Henning Tiemeier, Vilhelmina Ullemar, Marina Vafeiadi, Marinus H Van Ijzendoorn, Judith M Vonk, Annette Vriens, Martine Vrijheid, Pei Wang, Joseph L Wiemels, Allen J Wilcox, Rosalind J Wright, Cheng-Jian Xu, Zongli Xu, Ivana V Yang, Paul Yousefi, Hongmei Zhang, Weiming Zhang, Shanshan Zhao, Golareh Agha, Caroline L Relton, Vincent WV Jaddoe, Stephanie J London

**Affiliations:** 1 Department of Epidemiology, University Medical Center Rotterdam, Rotterdam, The Netherlands; 2 Department of Pediatrics, University Medical Center Rotterdam, Rotterdam, The Netherlands; 3 Generation R Study Group Erasmus MC, University Medical Center Rotterdam, Rotterdam, The Netherlands; 4 National Institute of Environmental Health Sciences, National Institutes of Health, Department of Health and Human Services, Research Triangle Park, USA; 5 Department of Environmental Health Sciences, Columbia University Mailman School of Public Health, New York, NY, USA; 6 MRC Integrative Epidemiology Unit, University of Bristol, Bristol, UK; 7 School of Oral and Dental Sciences, University of Bristol, Bristol, UK; 8 School of Social and Community Medicine, University of Bristol, Bristol, UK; 9 Department of Medical Epidemiology and Biostatistics, Karolinska Institutet, Stockholm, Sweden; 10 Pediatric Allergy and Pulmonology Unit at Astrid Lindgren Children’s Hospital, Karolinska University Hospital, Stockholm, Sweden; 11 Sorbonne Universités, UPMC Univ Paris 06, INSERM, Institut Pierre Louis d'Epidémiologie et de Santé Publique (IPLESP UMRS 1136), Epidemiology of Allergic and Respiratory diseases department (EPAR), Medical School Saint-Antoine, Paris, France; 12 Clinical and Experimental Sciences, Faculty of Medicine, University of Southampton, Southampton, UK; 13 Centre for Child and Family Studies, Leiden University, Leiden, The Netherlands; 14 Department of Epidemiology, School of Public Health, University of Michigan, Ann Arbor, USA; 15 Department Translational Research in Psychiatry, Max-Planck-Institute of Psychiatry, Munich, Germany; 16 Department of Psychiatry and Behavioral Sciences, Emory University School of Medicine, Atlanta, GA, USA; 17 Department of Biochemistry, Université de Sherbrooke, Sherbrooke, QC, Canada; 18 ECOGENE-21 and Lipid Clinic, Chicoutimi Hospital, Saguenay, QC, Canada; 19 Department of Preventive Medicine, Keck School of Medicine, University of Southern California, Los Angeles, USA; 20 Institute for Risk Assessment Sciences, Universiteit Utrecht, Utrecht, The Netherlands; 21 Julius Center for Health Sciences and Primary Care, University Medical Center Utrecht, Utrecht, The Netherlands; 22 Department of Pediatrics, Icahn School of Medicine at Mount Sinai, New York, NY, USA; 23 Department of Environmental Health, University of Cincinnati, Cincinnati, OH, USA; 24 Department of Medicine, University of California, San Francisco, CA, USA; 25 Department of Bioengineering and Therapeutic Sciences, University of California, San Francisco, CA, USA; 26 ISGlobal, Centre for Research in Environmental Epidemiology (CREAL), Barcelona, Spain; 27 Genomics and Disease Group, Bioinformatics and Genomics Program, Centre for Genomic Regulation (CRG), Barcelona, Spain; 28 Universitat Pompeu Fabra (UPF), Barcelona, Spain; 29 CIBER Epidemiología y Salud Pública (CIBERESP), Barcelona, Spain; 30 Department of Social Medicine, Faculty of Medicine, University of Crete, Heraklion, Greece; 31 Department of Pediatric and Adolescent Medicine, Oslo University Hospital, Oslo, Norway; 32 Norwegian Institute of Public Health, Oslo, Norway; 33 Department of Epidemiology, University Medical Center Groningen, University of Groningen, Groningen, the Netherlands; 34 Department of Epidemiology, Colorado School of Public Health; 35 Department of Pediatrics, University of Colorado Anschutz Medical Campus, Aurora, CO, USA; 36 Life Course Epidemiology of Adiposity and Diabetes (LEAD) Center, University of Colorado Anschutz Medical Campus, Aurora, CO, USA; 37 Environmental Risk and Health Unit, Flemish Institute for Technological Research (VITO), Mol, Belgium; 38 Faculty of Sciences, Hasselt University, Diepenbeek, Belgium; 39 The George Institute for Global Health, Nuffield Department of Obstetrics & Gynaecology, University of Oxford, Oxford, United Kingdom; 40 Center for Environmental Research on Children's Health, University of California, Berkeley, CA, USA; 41 Department of Environmental Health, Rollins School of Public Health, Emory University, Atlanta, USA; 42 Department of Mental Health, Bloomberg School of Public Health, Johns Hopkins University, Baltimore, USA; 43 Wendy Klag Center for Autism and Developmental Disabilities, Bloomberg School of Public Health, Johns Hopkins University, Baltimore, USA; 44 Department of Epidemiology, Regional Health Service, Lazio Region, Rome, Italy; 45 Instituto de Investigación Biosanitaria ibs. GRANADA, University of Granada, San Cecilio University Hospital, Granada, Spain; 46 Epigenetics Group, International Agency for Research on Cancer, Lyon, France; 47 Division of Chronic Disease Research Across the Lifecourse, Department of Population Medicine, Harvard Medical School and Harvard Pilgrim Health Care Institute, Boston, USA; 48 Department of Nutrition, Harvard T.H. Chan School of Public Health, Boston, MA, USA; 49 University of California, Berkeley, School of Public Health, Berkeley, USA; 50 Division of Metabolic and Nutritional Medicine, Dr. von Hauner Children’s Hospital, Ludwig-Maximilians Universität München (LMU), Munich, Germany; 51 Institute of Environmental Medicine, Karolinska Institutet, Stockholm, Sweden; 52 Department of Medicine, Université de Sherbrooke, Sherbrooke, QC, Canada; 53 Diabetes Unit, Massachusetts General Hospital, Boston, MA, USA; 54 Environmental Health Sciences Division, School of Public Health, University of California, Berkeley, CA, USA; 55 Human Development & Health, Faculty of Medicine, University of Southampton, Southampton, UK; 56 Department of Biological Sciences, North Carolina State University, Raleigh, NC, USA; 57 Center for Human Health and the Environment, North Carolina State University, Raleigh, NC, USA; 58 Telethon Kids Institute, University of Western Australia, Perth, WA, Australia; 59 Center For Lifecourse Health Research, University of Oulu, Oulu, Finland; 60 Biocenter Oulu, University of Oulu, Oulu, Finland; 61 Department of Epidemiology and Biostatistics, School of Public Health, Imperial College London, London, UK; 62 Bioinformatics Research Center, North Carolina State University, Raleigh, NC, USA; 63 Department of Preventive Medicine, Icahn School of Medicine at Mount Sinai, New York, NY, USA; 64 Mindich Child Health and Development Institute, Icahn School of Medicine at Mount Sinai, New York, NY, USA; 65 Department of Epidemiology, Geisel School of Medicine at Dartmouth, USA; 66 Children’s Environmental Health & Disease Prevention Research Center at Dartmouth, Hanover, NH, USA; 67 Division of Epidemiology, Biostatistics, and Environmental Health Sciences, School of Public Health, University of Memphis, Memphis, USA; 68 Department of Biostatistics and Informatics, Colorado School of Public Health, University of Colorado Anschutz Medical Campus, Aurora, USA; 69 Department of Biosciences and Nutrition, Karolinska Institutet, Stockholm, Sweden; 70 CIBER Epidemiología y Salud Pública (CIBERESP), Madrid, Spain; 71 IMIM (Hospital del Mar Medical Research Institute), Barcelona, Spain; 72 University of Groningen, Department of Pediatric Pulmonology and Pediatric Allergology, Beatrix Children's Hospital, GRIAC Research Institute Groningen, The Netherlands; 73 Department of Epidemiology, Bloomberg School of Public Health, Johns Hopkins University, Baltimore, USA; 74 Department of Psychology and Logopedics, Faulty of Medicine, University of Helsinki, Helsinki, Finland; 75 Collegium for Advanced Studies, University of Helsinki, Helsinki, Finland; 76 Department of Global Public Health and Primary Care, University of Bergen, Bergen, Norway; 77 Children's Hospital Colorado, Aurora, CO, USA; 78 Department for Non-Communicable Diseases, Domain for Mental and Physical Health, Norwegian Institute of Public Health, Oslo, Norway; 79 Department of Community and Family Medicine, Duke University Medical Center, Durham, NC, USA; 80 Sachs Children’s Hospital, Stockholm, Sweden; 81 Centre for Occupational and Environmental Medicine, Stockholm County Council, Stockholm, Sweden; 82 The Curtin UWA Centre for Genetic Origins of Health and Disease, Faculty of Health Sciences, Curtin University and Faculty of Medicine Dentistry & Health Sciences, The University of Western Australia, Perth, Australia; 83 Department of Obstetrics and Gynecology, Duke University Medical Center, Durham, NC, USA; 84 Centre for Environmental Sciences, Hasselt University, Diepenbeek, Belgium; 85 Department of Public Health & Primary Care, Leuven University, Leuven, Belgium; 86 National Center of Epidemiology, Surveillance and Health Promotion, Istituto Superiore di Sanità, Rome, Italy; 87 Research Unit for Gynaecology and Obstetrics, Department of Clinical Research, University of Southern Denmark, Odense, Denmark; 88 Department of Medical Sciences, University of Turin, Turin, Italy; 89 MRC/PHE Centre for Environment and Health School of Public Health, Imperial College London, London, UK; 90 Research Unit of Molecular Epidemiology, Institute of Epidemiology II, Helmholtz Zentrum Muenchen, Munich, Germany; 91 AOU Città della Salute e della Sceinza, CPO Piemonte, Turin, Italy; 92 Helen Diller Family Comprehensive Cancer Center (HDFCCC), UCSF, San Francisco, CA, USA; 93 Computational Biology Core, UCSF, San Francisco, CA, USA; 94 Department of Biomedical Sciences, University of Antwerp, Wilrijk, Belgium; 95 Department of Environmental Medicine, Institute of Public Health, University of Southern Denmark, Odense, Denmark; 96 Department of Immunology, University of Colorado Anschutz Medical Campus, Aurora, CO, USA; 97 Department of Medicine, University of Colorado Anschutz Medical Campus, Aurora, CO, USA; 98 Department of Genomics of Complex Diseases, School of Public Health, Imperial College London, London, United Kingdom; 99 Novo Nordisk Foundation Center for Basic Metabolic Research, Section on Metabolic Genetics, and Department of Public Health, Faculty of Health and Medical Sciences, University of Copenhagen, Copenhagen, Denmark; 100 Department of Clinical Epidemiology (formerly Institute of Preventive Medicine), Bispebjerg and Frederiksberg Hospital, The Capital Region, Copenhagen, Denmark; 101 National Institute of Environmental Health Sciences, Epidemiology Branch, Durham, NC, USA; 102 Department of Child and Adolescent Psychiatry, Erasmus MC, University Medical Center Rotterdam, Rotterdam, the Netherlands; 103 Department of Psychiatry, Erasmus MC, University Medical Center Rotterdam, Rotterdam, the Netherlands; 104 Department of Psychology, Education and Child Studies, Erasmus University Rotterdam, Rotterdam, The Netherlands; 105 University of Groningen, University Medical Center Groningen, Department of Epidemiology, GRIAC Research Institute Groningen, the Netherlands; 106 Department of Genetics and Genomic Sciences, Icahn School of Medicine at Mount Sinai, New York, NY, USA; 107 Icahn Institute for Genomics and Multiscale Biology, Icahn School of Medicine at Mount Sinai, New York, NY, USA; 108 Department of Epidemiology and Biostatistics, UCSF, San Francisco, CA, USA; 109 Department of Neurosurgery, UCSF, San Francisco, CA, USA; 110 Department of Pediatrics, Kravis Children's Hospital, Icahn School of Medicine at Mount Sinai, New York, NY, USA; 111 Mindich Child Health & Development Institute, Icahn School of Medicine at Mount Sinai, New York, NY, USA; 112 University of Groningen, University Medical Center Groningen, Department of Pulmonology, GRIAC Research Institute Groningen, the Netherlands; 113 University of Groningen, University Medical Center Groningen, Department of Genetics, Groningen, the Netherlands

## Why was the Consortium set up?

**Figure 1:**
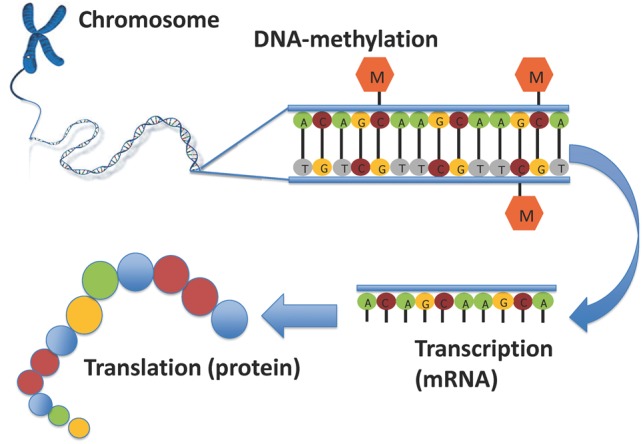
Schematic representation of DNA methylation. The figure shows a double DNA strand on the top right, with CpG sites which are methylated by the addition of a methyl group (M). DNA is transcribed into messenger RNA (mRNA). DNA methylation can influence transcription either positively or negatively, depending on the location of the methylated site. After transcription, mRNA is translated into proteins. Adapted with permission from Felix JF *et al.*^64^

Epigenetics refers to mitotically heritable changes to the DNA, which do not affect the DNA sequence, but can influence its function. Currently, DNA methylation is the most studied epigenetic phenomenon in large populations. It entails the binding of a methyl group, mainly to positions in genomic DNA where a cytosine is located next to a guanine, a cytosine-phosphate-guanine (CpG) site (Figure 1). DNA methylation at CpG sites can influence gene expression by altering the DNA’s three-dimensional structure and interacting with methyl-binding proteins, consequently affecting the binding of the gene transcription and chromatin-modifying machinery. There are approximately 28 million CpG sites in the human genome. DNA methylation is a dynamic process that can be influenced by genetic factors, as well as by environmental factors such as diet, air pollution, toxicants or smoking.^1–4^ Hence, DNA methylation may be seen as linking the genome to the environment with respect to health and disease. Early development is a period of profound changes in DNA methylation and may, as such, be a critical period for environmentally-induced DNA methylation changes.^4^ Hence, this period is of specific interest for DNA methylation studies in relation to specific exposures and long-term health outcomes.^1^^,^^4–6^

DNA methylation modifications in early life represent an important potential mechanism for studies on the developmental origins of health and disease (DOHaD). The DOHaD hypothesis suggests that exposure to an adverse environment in fetal life or early childhood leads to permanent changes in organ structure or function, which may have effects on later life health.^7^^,^^8^ Many associations of early life adverse exposures, such as maternal obesity, smoking, air pollution and suboptimal diet, with common diseases throughout the life course have been described.^9–12^ Long-lasting DNA methylation modifications may be an important mechanism linking early life exposures with outcomes in later life.^13^ Besides having a potential mechanistic role, DNA methylation may also serve as a biomarker of exposures or outcomes, even without it having a direct causal role in the process.^3^^,^^14^^,^^15^ For example, an environmental factor may cause both a change in phenotype and a change in DNA methylation, without a causal relation between the two. Also, a disease could cause a change in DNA methylation, rather than the other way around.^15^ The ability of methylation signals to serve as strong biomarkers of some exposures, such as maternal smoking in pregnancy, may complicate inference about the role in mediating health outcomes; measurement error correction may help in this regard.^16^ Various pregnancy, birth and childhood studies have recently initiated research on the role of DNA methylation in the response to environmental exposures and development of health outcomes. Individual studies usually have sample sizes too small to address this issue, but it can be studied in joint efforts of prospective cohort studies starting from early life onwards.^1^^,^^17^

The potential of collaborative efforts between large-scale prospective cohort studies has been demonstrated by the success of recent genome-wide association studies (GWAS) which have shed light on the genetic background of common diseases as well as their risk factors. These GWAS are characterized by state-of-the-art genome-wide agnostic approaches in which millions of genetic variants are related to a particular health outcome, usually in the setting of large consortia combining the results of multiple studies, using meta-analysis. Common genetic variants have been identified that are related to birthweight, childhood obesity, respiratory phenotypes, atopic dermatitis and behavioural outcomes among others.^18–25^ In line with these approaches, recent developments enable analysis of hundreds of thousands of DNA methylation markers across the genome on a single array.^26^^,^^27^ The high-throughput and cost-effective nature of these arrays has made it possible for studies to measure DNA methylation across the genome (‘epigenome-wide DNA methylation’) in relatively large samples sizes. These data can be used in epigenome-wide association studies (EWAS) to evaluate associations of DNA methylation at specific sites or regions of the genome with determinants and outcomes of health and disease. EWAS in pregnancy, birth or child cohorts specifically enable exploration of associations of early life exposures with DNA methylation levels in children, and of DNA methylation levels with specific growth, development and health outcomes. Recent study-specific EWAS have shown associations of DNA methylation levels in offspring with birthweight, maternal body mass index and maternal smoking.^28–31^ Large sample sizes are required to achieve optimal power in analyses of so many genomic sites, especially if the prevalence of the exposure or outcome under study is low. Collaboration between studies and combined meta-analysis of the available data are needed to optimize the use of resources and to increase the likelihood of detecting DNA methylation differences underlying the associations of early life exposures and health outcomes.

This paper describes the global Pregnancy And Childhood Epigenetics (PACE) Consortium which, to date, brings together 39 studies with over 29 000 samples and DNA methylation data in pregnant women, newborns and/or children. Besides strongly increased power to detect associations, bringing studies together in the PACE Consortium for meta-analysis greatly decreases the risk of false-positive associations. The larger power also enables more detailed studies into potential causal roles of methylation, using a mendelian randomization approach for which large sample sizes are typically needed. In addition, a number of studies have measured DNA methylation at multiple time points from birth through childhood and/or in adolescence, which enables investigation into the persistence of differential DNA methylation signals over time. Also, the availability of information from studies with participants from various backgrounds in terms of ethnicity, location and living environment enables testing of identified associations across different settings and evaluation of heterogeneity of effects across study populations.

The primary aim of the PACE Consortium is to identify differences in DNA methylation in relation to a wide range of exposures and outcomes pertinent to health in pregnancy and childhood through joint analysis of DNA methylation data. Secondary aims of the Consortium are to perform further functional annotation-based analyses, to attempt to assess causality of DNA methylation differences for child health phenotypes, to contribute to methodological development and to exchange knowledge and skills.

## Who is in the Consortium?

In June 2013, an international group of studies focused on maternal and child health met at the U.S. National Institute of Environmental Health Sciences to organize an EWAS meta-analysis on maternal smoking in pregnancy and DNA methylation in newborns and children.^32^ This marked the start of the PACE Consortium. The success of this initial effort resulted in the expansion of the Consortium and inclusion of additional research groups, to include additional exposures and outcomes. The PACE Consortium is modelled after successful GWAS consortia, in which many PACE investigators already participated, including the Early Growth Genetics (EGG) Consortium, the Early Genetics and Lifecourse Epidemiology (EAGLE) Consortium and the Cohorts for Heart and Aging Research in Genomic Epidemiology (CHARGE) Consortium.^33^ Currently, the PACE Consortium includes 39 studies with genome-wide DNA methylation data from pregnancy, newborn or childhood samples and information on at least one of the exposures or outcomes of interest. A list of studies currently involved in the PACE Consortium with basic study information is shown in Table 1. More detailed descriptions of the individual cohorts can be found in the Supplementary material and Supplementary Table 1, available at *IJE* online. The PACE Consortium is an open, dynamic collaboration and additional research groups are welcome to join.

**Table 1:** List of studies currently involved in the PACE Consortium with basic study information

Study	Study reference (PMID)	Study website	Design of base study^a^	Country	Year(s) of birth of base study^a^	Total *N* of base study^a^	Ethnicity	Sex, % female	Selection criteria for EWAS
**ALSPAC**	22507743, 25991711	http://www.bristol.ac.uk/alspac/	Population-based birth cohort	UK	1991–92	14 541	96.1% European	49.7	Selected based on availability of DNA samples at two time points for the mother and three time points for the offspring
**BAMSE**	12688617, 27040690, 20860503, 23517042	http://ki.se/en/imm/bamse-project	Population-based birth cohort	Sweden	1994–96	4089	>95% European	49.5	European, asthma cases and controls
**CBC**	27403598, 26646725	http://circle.berkeley.edu	Nested case-control within a population birth cohort	USA	1982–2009	1200	Hispanic, non-Hispanic White, non-Hispanic others (African Americans, Asians, other and mixed ethnicity)	40	Adequate exposure data
**CHAMACOS**	15238287, 23959097, 16203258	http://cerch.berkeley.edu/research-programs/chamacos-study	Population-based birth cohort	USA	1999–2000	601	Mexican Americans	50 (birth); 60 (2 y); 60 (5 y); 54 (9 y); 0 (12 y)	Repeat sampling of the same children
**CHOP**	19386747, 24622805, 27171005, 25368978	NA	Intervention study and birth cohort	Belgium, Germany, Spain, Italy, Poland	2002–04	1678	European	49.3	Selected based on availability of DNA samples
**CHS**	16675435, 22896588	https://healthstudy.usc.edu/index.php	Population-based cohort	USA	1995–97	5341	4% Asian; 4% Black; 35% non-Hispanic White; 55% Hispanic White	49	Non-Hispanic White or Hispanic White, GWAS availability, availability of air pollution exposure assessment and cardiorespiratory measures in follow-up
**EARLI**	22958474	http://www.earlistudy.org/	Enriched autism risk pregnancy cohort	USA	2009–12	232	60% European; 8% Black; 8% Asian; 24% Other	47.4	NA
**EDEN**	26283636	http://eden.vjf.inserm.fr/index.php/fr/	Population-based birth cohort, enrolled before 24 weeks of pregnancy	France	2003–06	2002	European	47.4	European, complete follow-up
**ENVIR*ON*AGE**	23742113	www.limburgsgeboortecohort.be	Population-based birth cohort	Belgium	2010–16	1210	86% European	49	Random sample; questionnaire at birth available and availability of cord blood samples and placenta tissue
**EPOCH**	23741625, 22508709, 22290537, 21238981, 20953862	NA	Historical prospective cohort of GDM exposed and unexposed offspring	USA	200510	604	56% non-Hispanic White; 33% Hispanic; 11% other	46	All exposed to GDM; 1:1 matched sample of unexposed
**FLEHS I**	28160993, 19539994	http://www.milieu-en-gezondheid.be/English/index.html	Population-based birth cohort	Belgium	2002–04	1196	European	48	Selected based on availability of DNA samples at two time points for the children; i.e. at birth from cord blood and at 11 years from blood and saliva
**GALA II**	23684070, 23750510	http://pharm.ucsf.edu/burchard/research/study-populations	Case-control	USA	Aged 8–21 at recruitment; recruited 2006–11	4157	Latino	50	Random sample
**GECKO**	18238823	www.geckodrenthe.umcg.nl	Population-based birth cohort	The Netherlands	2006–07	2874	95% European; 5% mixed	49.7	Case-control (cases: intrauterine smoke exposure) and complete follow-up
**Generation R**	28070760, 25527369	www.generationr.nl	Prospective population-based birth cohort	The Netherlands	2002–06	9901	50% European; 50% mixed other	49.3	European, complete follow-up
**Gen3G**	26842272	NA	Population-based birth cohort	Canada	2010–13	1034	95% European	48	Complete data during pregnancy and paired placenta + cord blood samples
**GOYA**	21935397	www.dnbc.dk	Case-cohort sample of the Danish National Birth Cohort, which is a population-based birth cohort	Denmark	1996–2002	91 387 (DNBC) 3908 (GOYA)	European	49	1000 children equally sampled from extreme obese GOYA mothers (cases) and GOYA control mothers
**Healthy Start**	27133623, 26872289, 26663829, 26055075, 25646327, 25628236, 25574704	http://www.ucdenver.edu/academics/colleges/PublicHealth/research/ResearchProjects/Pages/healthystart.aspx	Pre-birth cohort	USA	2009–14	1410	53% non-Hispanic White; 24% Hispanic; 17% non-Hispanic Black; 6% other	51	Available cord blood DNA, maternal serum and urine
**ICAC/EPIGEN**	25769910, 27745942	http://www.rhoworld.com/rho/services/projects/icac	Allergic asthma case-control	USA	1998–2005	200	100% African American	50	High quality DNA and RNA samples
**INMA**	21471022	http://www.proyectoinma.org/	Population-based birth cohort	Spain	1997–2008	3768	>90% European	39	Blood: available DNA from one of the subcohorts (Sabadell); placenta: selection of children with detailed information on exposures from 4 subcohorts (Sabadell, Gipuzkoa, Valencia and Asturias)
**IoW F1 Generation**	22607991, 28183434	www.allergyresearch.org.uk/	Prospective cohort	UK	1989–90	1456	European	49	Random sample ∼2:1 F:M ratio of subjects with biological samples available at age 18
**IoW F2 Generation**	26199674, 28183434	www.allergyresearch.org.uk/	Prospective cohort	UK	2012–17	420 (recruiting is continuing)	European	44,3	Recruited at birth with cord blood samples available
**MoBa 1**	27063603, 17031521, 27040690	https://www.fhi.no/en/studies/moba/	Population-based pregnancy cohort	Norway	1999–2009	114479	European	48.7	Asthma at 3 y plus cohort random sample
**MoBa 2**	27063603, 17031521, 27040690	https://www.fhi.no/en/studies/moba/	Population-based pregnancy cohort	Norway	1999–2009	114479	European	48.7	Asthma at 7 y, random noncases, cohort random sample
**MoBa 3**	27063603, 17031521, 27040690	https://www.fhi.no/en/studies/moba/	Population-based pregnancy cohort	Norway	1999–2009	114479	European	48.7	Case-control (childhood cancer and 2 controls per case matched on birth year only)
**NCL**	17259187, 24906187	https://www.niehs.nih.gov/research/atniehs/labs/epi/studies/ncl/	National population-based case-control study of cleft lip and cleft palate	Norway	1996–2001	1336	European	43	Random sample
**NEST**	21255390, 21636975	https://sites.duke.edu/nest/	Population-based birth cohort	USA	2005–09	895 women (936 mother-child pairs)	53% African American; 43% European; 4% other	49.5	Follow-up height and weight data available
**NFBC 1966**	NA	www.oulu.fi/nfbc	Population-based birth cohort	Finland	1966	12 231	European	50	Random sample
**NFBC 1986**	NA	www.oulu.fi/nfbc	Population-based birth cohort	Finland	1985–86	9362	European	50	Random sample
**NHBCS**	26771251, 26955061, 26359651, 23757598	https://www.dartmouth.edu/∼childrenshealth/scientists.php	Prospective longitudinal pregnancy cohort	USA	2009 ongoing	1500	Mostly European	48.8	Time-delimited sample with complete data
**PIAMA**	23315435, 12688620	piama.iras.uu.nl	Population-based birth cohort	The Netherlands	1996–97	3963	European	48.2	European; 4 y and 8 y for MeDALL asthma study; 16 y general population
**Piccoli+**	24506846	www.piccolipiu.it	Population-based birth cohort	Italy	2011–15	3338	Mainly European	48.7	Random sample; resident in Turin, with growth data until at least 2 years of age and availability of cord blood samples
**PREDO**	27639277	NA	Birth cohort	Finland	2006–10	1079	European	47	NA
**PRISM**	24476840, 25328835	NA	Population-based prenatal cohort	USA	2012–14	592	38% European; 39% Black/Haitian; 13% Hispanic; 10% other/mixed	46	Random sample
**Project Viva**	24639442	https://www.hms.harvard.edu/viva/index.html	Longitudinal pre-birth cohort	USA	1999–2003	2128	Maternal: 66.5% White; 16.5% Black; 7.3% Hispanic; 5.7% Asian; 3.9% other	48.5	Available venous cord blood or early childhood or mid-childhood blood sample, and genetic consent
**Raine**	8105165, 26169918	http://www.rainestudy.org.au/	Population-based pregnancy cohort	Australia	1989–91	2868	88.3% European; 2.3% Aboriginal; 9.4% other	48.6	DNA collected at 1-year-old follow up
**Rhea**	19713286	www.rhea.gr	Population-based birth cohort	Greece	2007–08	1500	Mainly European (91% Greek)	49.6	Random sample; resident in Heraklion region with cord blood and complete follow-up and clinical evaluation at 4 years
**RICHS**	27004434	NA	Population-based birth case-cohort	USA	2009–14	840	Mostly European	50.3	Time-delimited sample with complete data
**SEED I**	22350336	https://www.cdc.gov/ncbddd/autism/seed.html	Autism case-control	USA	2003–06	3899	56.4% European; 12% Black; 3.8% Asian; 25.2% admixed	33	Autism case or population control
**STOPPA**	25900604	http://ki.se/meb/stoppa	Population-based twin cohort	Sweden	1997–2004	752	Mostly European	47	All with blood samples available

NA, not available; y, years; GDM, gestational diabetes mellitus; F, female; M, male.

^a^Base study refers to the underlying study population from which the EWAS subjects came.

The Consortium structure is purposefully kept simple. The work in the Consortium is strongly researcher-driven. Any member can propose an analysis. Projects are often co-led by two or more researchers from different studies. This supports collaboration and exchange of knowledge and skills for both junior and senior researchers. On most projects, junior researchers, often PhD students or postdoctoral students, take the lead under the supervision of a more experienced, senior researcher from their own or another participating institution. The lead group operates as the meta-analysis centre for a specific project. For each project, a working group is formed and studies can opt into or opt out of that specific project. Analyses are performed according to a predefined analysis plan, which contains inclusion and exclusion criteria, phenotype definitions, covariates and statistical models, usually logistic or robust linear regression models. Each cohort performs its own quality control and normalization of the EWAS data. We have shown a very limited influence of different normalization methods between cohorts on the results of EWAS meta-analyses.^32^ Each cohort analyses its own data according to the analysis plan, after which the summary results are shared with the meta-analysis centre. Data exchange is organized for each project separately, usually through secure university-based upload servers. These summary results include the effect estimate, standard error, *P*-value and included sample size for each CpG analysed. In general, meta-analysis of summary results is the preferred approach and no individual-level data are shared between the centres. However, integrated data approaches may be considered, conditional on ethical and legal agreements, which may differ for each individual study; but such approaches have not been used so far. Subsequently, the meta-analysis centre performs quality control of the summary results files and meta-analyses all datasets, with specific ‘omics’ meta-analysis software, such as Metal.^34^ Standard quality controls include inspection of the distribution of effect estimates and standard errors across cohorts, and Manhattan plots of individual cohort and meta-analysis results. The full process of quality control and meta-analysis is independently repeated by an analyst from one of the other participating studies (the ‘second centre’) as a quality control measure.

**Figure 2:**
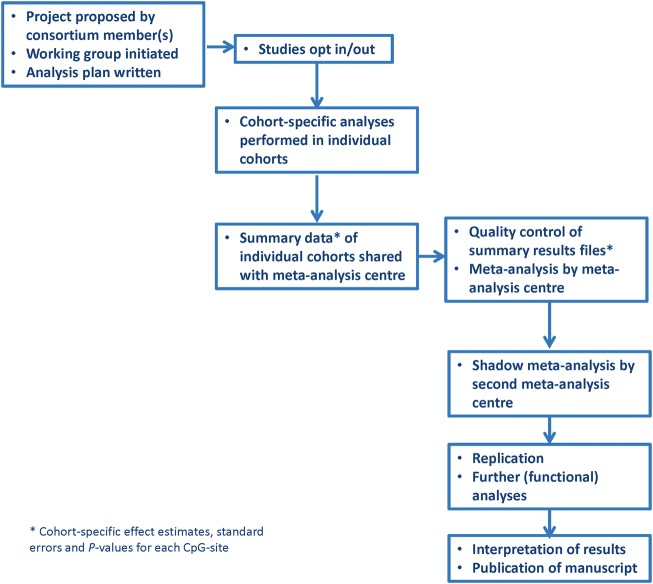
Flow diagram of the analytical processes.

As a general rule, as many studies as possible are included in the discovery meta-analysis to increase power to discover new associated DNA methylation sites. Replication of findings is then pursued in further studies that were unable to participate in the discovery meta-analysis, if available. After the discovery meta-analysis is finished, further work is done in terms of validation and interpretation of the results, including enrichment/pathway/functional network analyses using publicly available resources, and methylation-expression analyses (Figure 2). Often, such follow-up work involves a look-up of the main findings in children of different ages than in the main analysis. For example, after a discovery analysis in cord blood samples, a look-up of the findings in childhood and adolescent samples may be done to study persistence of the identified signals.

Analyses in the PACE Consortium are performed collaboratively by the participating centres. Logistics are organized by the National Institute of Environmental Health Sciences in Research Triangle Park, NC, USA. All ongoing and proposed analyses are discussed in bi-weekly conference calls, during which project leaders give updates. In addition, individual analysis groups may have separate conference calls if needed. 

## How often have they been followed-up?

The PACE Consortium brings together a large number of cohorts, each of them with cohort-specific protocols (Table 1, Supplementary Table 1). Most studies have ongoing data collection and follow-up. Many of the cohorts have multiple follow-up time points from fetal life into childhood, and several have follow-up into adolescence or early adulthood. Most have information on maternal exposures during pregnancy, including maternal smoking and body mass index.^31^^,^^32^ A number of studies also collected information on more specific exposures, such as air pollution.^35^ All cohorts have collected information on child physical and/or mental development. Some studies have a particular focus, such as cleft lip and palate (NCL) or autism (SEED I), but most are population-based cohorts collecting a vast amount of data on many domains. These include anthropometric, cardiometabolic, neurodevelopmental and respiratory measurements, as well as childhood diseases. Further details of data collection waves, follow-up and biological sample collection in all studies can be found in Supplementary Table 1. The PACE Consortium is focused around the common methylation platform. Studies commit to the PACE Consortium on a project-by-project basis. They are not necessarily involved in PACE with all their data, but rather decide per project whether or not they will participate. It is therefore possible that a particular study is not involved in a PACE project on a specific topic, for example because they decide to pursue a single-study project or because they are involved in another collaboration on that topic. In such cases, studies opt out of the project and are not involved until the work is published. With ongoing sample collection and data expansion in each study, increased DNA methylation and phenotype measures will be available in the future. Multiple cohorts have longitudinal measurements of DNA methylation, and investigations on the persistence of DNA methylation signals are possible. Repeated measurements of outcomes during childhood, adolescence and beyond will enable specific developmental or life course trajectory analyses in relation to DNA methylation signals. Study-specific details are described in the Supplementary material and Supplementary Table 1.

## What has been measured?

**Figure 3:**
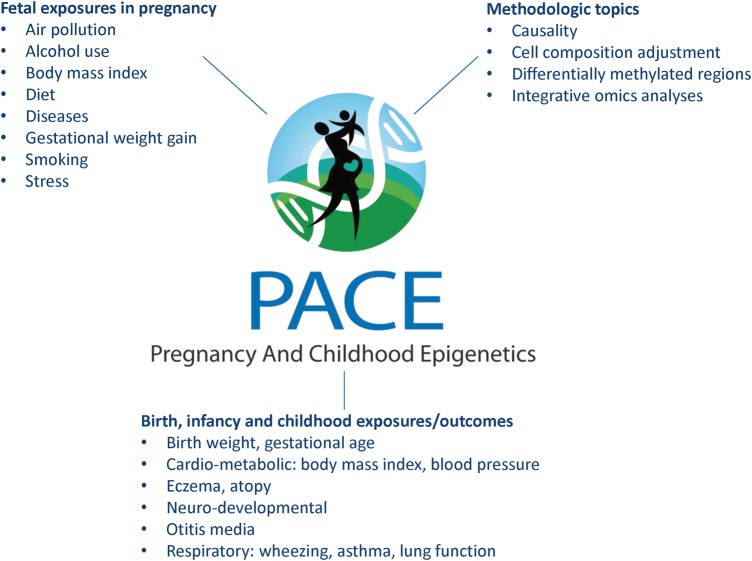
Current main exposures, outcomes and methodological topics in the PACE Consortium.

All studies involved in the PACE Consortium have common measures of DNA methylation. Currently the platform used by the group is the Illumina 450 K HumanMethylation array, the most widely used array in large-scale human studies (Illumina Inc., San Diego, USA).^26^ Recently a larger, compatible array (850 K EPIC) was developed.^27^ New studies using this array can be included in the Consortium in the future. The 450 K array includes around 485 000 DNA methylation sites, covering less than 2% of all sites across the genome. It is targeted at genes and CpG islands, and sites were chosen based on advice of an international group of DNA methylation experts.^26^ The PACE Consortium currently focuses on exposures occurring during pregnancy and childhood health outcomes. Across studies, a vast number of exposures and outcomes are available and studies usually participate in multiple analyses. The main exposures that the PACE Consortium currently focuses on are those occurring during pregnancy; the main outcomes are childhood health parameters and diseases. An overview is given in Figure 3. Recently, working groups have formed around methodological issues, such as blood cell composition adjustment and evaluation of methods for identifying differentially methylated regions. Many of the studies involved in the PACE Consortium also have GWAS data and other types of ‘omics’ data, including transcriptomics and metabolomics if available, creating the possibility for integrative omics analyses. The availability of GWAS data enables analyses of associations of genetic variants with DNA methylation, as well as analyses to assess the potential influence of genetic variation on methylation variance, the possible causal role of DNA methylation differences using a two-step mendelian randomization approach, and adjustment for genetic markers of ancestry.^36–38^

## What has it found?

A number of the cohorts involved in the PACE Consortium have published cohort-specific EWAS on various phenotypes, including maternal smoking, maternal body mass index, maternal stress and child birthweight and sex, pre-dating PACE projects on these topics.^28–31^^,^^39–43^ Some studies have involved collaborations between a few of the PACE cohorts.^44–47^ In addition, members of the PACE Consortium have contributed to methodological developments in the field, such as evaluation of normalization methods, aspects of study design, and analysis software development.^48–54^ Multiple consortium projects are currently being analysed or prepared. Here, we would like to highlight the first three published reports.

The first large PACE Consortium meta-analysis reported on the results of a meta-analysis on maternal smoking in relation to cord blood DNA methylation.^32^ This meta-analysis of EWAS was on sustained maternal smoking during pregnancy in 13 cohorts, with a total of 6685 newborns. There were 6073 differentially methylated CpG sites in relation to maternal smoking during pregnancy, after multiple testing correction using a false discovery rate of 5%, of which half had not previously been identified for their association with either maternal smoking during pregnancy or smoking in adults. This analysis showed the increased power leveraged by large consortium analysis. Analyses of older children (five cohorts, *N* = 3187) indicated that most of these DNA methylation signals observed at birth persist into childhood, but are attenuated. A number of the differentially methylated CpG sites were in or near genes with known roles in diseases associated with maternal smoking, such as orofacial clefts and asthma. We also found enrichment in developmental processes.

The second report was a meta-analysis of the association of maternal plasma folate levels during pregnancy among 1988 newborns from two cohorts. Differential methylation of 443 CpG sites related to 320 genes was found, with most of these genes having no known function in folate biology.^44^

The third, most recent meta-analysis reported the results of an assessment of the association of prenatal air pollution exposure and cord blood DNA methylation in four cohorts, spanning 1508 participants.^55^ It showed that exposure to nitrogen dioxide during pregnancy was associated with differential offspring DNA methylation in mitochondria-related genes, as well as in several genes involved in antioxidant defence pathways. Some of these associations also persisted to older ages.^55^ A current overview of published papers from the PACE Consortium can be found at: [http://www.niehs.nih.gov/research/atniehs/labs/epi/pi/genetics/pace/index.cfm].

## What are the main strengths and weaknesses?

### Main strengths

Although individual-cohort analyses can reveal associated DNA methylation sites, joining forces in meta-analyses within a consortium brings significant benefits. First, it substantially increases sample size, facilitating the discovery of novel loci and optimizing the use of resources. Second, it offers the potential for analyses of DNA methylation signals at various ages throughout infancy, childhood and adolescence. Third, this setting makes it possible to compare effects between different populations and ethnicities. Fourth, a consortium setting allows replication of findings across studies, thus decreasing the publication of false-positive results from individual studies. Fifth, EWAS analyses in pregnancy, birth and child cohort studies offer an enormous potential to shed light on mechanisms underlying the associations of early, fetal and childhood exposures with later life health and disease, and on a potential role of DNA methylation as a biomarker of exposures or outcomes. The longitudinal data collection from early life onwards enables us to study the role of DNA methylation in life course health trajectories. Sixth, the experience and diverse backgrounds of the PACE investigators, including epidemiologists, statisticians, geneticists, clinicians, bioinformaticians and biologists, enables sharing of methods and analytical code, quicker solutions to methodological issues and easier exchange of knowledge and skills. The experience of many PACE investigators in existing consortia, often with the same partner studies, was of great benefit at the start of the PACE Consortium. Issues that may have posed challenges to earlier consortia, such as communication between studies, harmonizing analytical methods, and authorship strategies, were hence part of the ‘basic skill set’ of this Consortium.^33^ Seventh, the Consortium also offers outstanding networking opportunities for students, postdocs and junior investigators in their career development. Based on recent experience in GWAS consortia, we expect that the PACE structure can be a springboard for both junior and senior investigators to apply for funding for new projects, including those that require additional analyses of samples, exposures or outcomes. Similar to many other consortia, the PACE Consortium has no structural or central funding other than the modest administrative support from the National Institute of Environmental Health Sciences for conference calls, the website and the three in-person meetings held to date.

### Main weaknesses

Analyses of epigenome-wide DNA methylation face particular methodological challenges. First, the analyses in the PACE Consortium are mainly performed on DNA extracted from blood samples, which are easily collected in population-based settings. However, each cell type may have its own unique methylation profile. Thus, DNA methylation in leukocytes does not necessarily represent DNA methylation in other tissues that may be more relevant for certain phenotypes, for example lung tissue when studying the association of DNA methylation and asthma. This feature of DNA methylation studies in blood poses a challenge in the interpretation of the findings. As cohort studies involving young children will generally not be able to collect more specific tissue samples, with the exception of buccal cells, collaborations will be sought with other partners in the future to be able to address tissue specificity. A subset of PACE cohorts have DNA methylation measured in placenta. Second, the distribution of blood cell subtypes in blood samples varies in response to a range of internal and external factors, such as infection, diseases and smoking. As DNA methylation is cell-type specific, an observed association of an exposure or an outcome with DNA methylation may be the result of changes in blood cell composition, rather than a representation of a true association. Adjustment for blood cell composition in studies using cord blood data is a challenge. So far, we have used the regression calibration method of Houseman and colleagues, which until recently has been constrained to the first available reference panel of 450 K data in white blood cell subtypes of six adult males. This panel has been shown to be suboptimal in estimating blood cell proportions in DNA from newborns.^49^^,^^56^^,^^57^ Recently, PACE consortium investigators reported on cord blood-specific methods for blood cell composition correction.^49^^,^^58^^,^^59^ Third, as in any epidemiological study, but less problematic in GWAS, confounding factors need to be taken into account in the analyses. In addition, confounding by technical covariates, or batch effects, which has minimal effect on genotype calling in GWAS, needs to be addressed in EWAS and may require extensive adjustment. Given the size of the Consortium and the number of studies that may be involved in a meta-analysis, it can also be a challenge in terms of logistics and time to ask individual studies to go back and re-run analyses with additional covariates or stratified on a particular factor such as sex to study associations in more detail. Fourth, as certain outcomes or disease states may also influence DNA methylation, the potential for reverse causality needs to be taken into account, especially in cross-sectional analyses. Yet, even if a disease causes differences in DNA methylation, these may still serve a clinical purpose as biomarker of the disease or its progression.^15^ Such epigenetic biomarkers may be used in disease prediction, as a diagnostic test, in determining specific disease subtypes or in informing on prognosis.^3^ Fifth, the currently used DNA methylation arrays only cover 2–3% of the total number of DNA methylation sites, with a focus on genes and CpG islands.^26^^,^^27^ Even though the newer EPIC array increases coverage of enhancer regions, the coverage will still be relatively limited.^27^ Sixth, the integration of DNA methylation data with other ‘omics’ data to gain insight into their interrelations will also pose challenges, both in terms of methodology and in terms of bioinformatics approaches. An in-depth discussion of these methodological challenges is beyond the scope of this article, but these are topics of ongoing work within and outside the PACE Consortium.^50^^,^^60–63^ Seventh, the studies currently involved in the PACE Consortium are located in industrialized countries. Studying environmental exposures in low- and middle-income settings would be relevant for a more complete understanding of epigenetic mechanisms. As PACE is an open consortium, we hope to be able to include studies from developing countries in the future.

There is much to learn in the field of EWAS. The efforts by this Consortium and many other researchers represent the first steps in the discovery of the role of DNA methylation in health and disease. Results from EWAS meta-analyses do not stand on their own. Discovery results from EWAs need to be followed by investigation of the relationships between DNA methylation and gene expression, of the roles of biological pathways on outcomes and of causality between exposures and DNA methylation. Conversely, results from laboratory scientists may inspire new analyses of DNA methylation in human studies. Many methodological issues need to be resolved. The PACE Consortium offers a strong platform to address these points and to contribute to the field of population epigenetics in the future.

## Can I get hold of the data? Where can I find out more?

The PACE Consortium is an open consortium and studies interested in participating in one or more analyses are welcome to join. Each individual cohort analyses its own data locally and only summary statistics, including cohort-specific effect estimates, standard errors and *P*-values for each CpG site, are shared for the meta-analysis. Therefore, for access to data from individual cohorts in the PACE Consortium, researchers should contact studies directly. Study-specific protocols can be found through the study websites (Supplementary material and Supplementary Table 1) or through contact with study investigators. Researchers interested in participating in the PACE Consortium can contact the corresponding authors of this paper. Meta-analysis summary statistics will be made publicly available, in accordance with journal requirements. For more information, please see: [http://www.niehs.nih.gov/research/atniehs/labs/epi/pi/genetics/pace/index.cfm].

## Supplementary Data

Supplementary data are available at *IJE* online.

Profile in a nutshell

- The PACE Consortium is an open consortium that brings together studies with epigenome-wide DNA methylation data in pregnant women, newborns and/or children, with the aim to identify, using meta-analysis, differences in DNA methylation in association with a wide range of exposures and outcomes related to health across the life course.
- Currently, the consortium includes 39 studies with over 29 000 samples with epigenome-wide DNA methylation data. Participation is on a project-by-project basis.
- Projects to date include gestational exposures and maternal behaviours, such as maternal alcohol use, body mass index, gestational weight gain, stress, diet, air pollution, maternal diseases and smoking. Outcomes under study include childhood growth and obesity, and cardiometabolic, neurodevelopmental, respiratory and allergic phenotypes.
- Researchers interested in participating can contact the corresponding authors (J.F.F. and S.J.L.) of this paper. For more information: [http://www.niehs.nih.gov/research/atniehs/labs/epi/pi/genetics/pace/index.cfm].

## Funding

### Avon Longitudinal Study of Parents And Children (ALSPAC)

The UK Medical Research Council and the Wellcome Trust (grant ref: 102215/2/13/2) and the University of Bristol provide core support for ALSPAC. The Accessible Resource for Integrated Epigenomics Studies (ARIES), which generated large-scale methylation data, was funded by the UK Biotechnology and Biological Sciences Research Council (BB/I025751/1 and BB/I025263/1). Additional epigenetic profiling on the ALSPAC cohort was supported by the UK Medical Research Council Integrative Epidemiology Unit and the University of Bristol (MC_UU_12013_1, MC_UU_12013_2, MC_UU_12013_5 and MC_UU_12013_8), the Wellcome Trust (WT088806) and the United States National Institute of Diabetes and Digestive and Kidney Diseases (R01 DK10324). The funders had no role in study design, data collection and analysis, decision to publish or preparation of the manuscript.

### Children’s Allergy Environment Stockholm Epidemiology study (BAMSE)

The BAMSE cohort was supported by the Swedish Research Council, the Swedish Heart-Lung Foundation, Freemason Child House Foundation in Stockholm, MeDALL (Mechanisms of the Development of ALLergy), a collaborative project conducted within the European Union (grant agreement No. 261357), Stockholm County Council (ALF), Swedish Foundation for Strategic Research (SSF, RBc08–0027, EpiGene project), the Strategic Research Programme (SFO) in Epidemiology at Karolinska Institutet, the Swedish Research Council Formas and the Swedish Environment Protection Agency.

### California Birth Cohort (CBC)

Funding provided to J.L.W. by the Center for Integrative Research on Childhood Leukemia and the Environment (P01ES018172), NIH grants P50ES018172 and R01ES09137, EPA RD83451101, RD83615901, and NIH 5P30CA082103 (the UCSF Comprehensive Cancer Center Support grant). R.R. is supported by P30 CA82103 (the UCSF Comprehensive Cancer Center Support grant). S.G. is supported by the Swiss Science National Foundation [grants number: P2LAP3_158674] and the Sutter-Stöttner Foundation.

### Center for Health Assessment of Mothers and Children of Salinas (CHAMACOS)

The CHAMACOS study was supported by the NIH grants P01 ES009605 and R01 ES021369, R01ES023067 and EPA grants RD 82670901 and RD 83451301.

### Childhood Obesity Project (CHOP)

The CHOP study and research reported herein were partially supported by: the Commission of the European Community, specific RTD Programme ‘Quality of Life and Management of Living Resources’ within the 5th Framework Programme (research grant nos. QLRT-2001–00389 and QLK1-CT-2002–30582); the 6th Framework Programme (contract no. 007036); the European Union’s Seventh Framework Programme (FP7/2007–2013), project EarlyNutrition under grant agreement no. 289346; and the European Research Council Advanced grant ERC-2012-AdG – no.322605 META-GROWTH. This manuscript does not necessarily reflect the views of the Commission and in no way anticipates the future policy in this area. The funders had no role in study design, data collection and analysis, decision to publish or preparation of the manuscript.

### Children’s Health Study (CHS)

The CHS was supported by the following NIH grants: K01ES017801, R01ES022216, P30ES007048, R01ES014447, P01ES009581, R826708–01 and RD831861–01. C.V.B. has received funding from NIH grants P50ES026086, R01ES022216, K01ES017801 and EPA grant 83615801–0.

### Early Autism Risk Longitudinal Investigation cohort (EARLI)

Funding for this work was provided by R01ES017646, R01ES01900, R01ES16443, and Autism Speaks grant #260377.

### Etudes des Déterminants pré et postnatals précoces du développement et de la santé de l’Enfant (EDEN)

EDEN funding was provided by: Funds for Research in Respiratory Health, the French Ministry of Research: IFR program, INSERM Nutrition Research Program, French Ministry of Health: Perinatality Program, French National Institute for Population Health Surveillance (INVS), Paris–Sud University, French National Institute for Health Education (INPES), Nestlé, Mutuelle Générale de l’Education Nationale (MGEN), French-speaking association for the study of diabetes and metabolism (Alfediam), grant # 2012/51290–6 Sao Paulo Research Foundation (FAPESP), EU-funded MedAll project.

### ENVIRonmental influence ON early AGEing (ENVIRonAGE)

The ENVIRonAGE birth cohort is funded by the European Research Council (ERC-2012-StG.310898) and by funds of the Flemish Scientific Research Council (FWO, N1516112 / G.0.873.11 N.10). The methylation assays were funded by the European Community's Seventh Framework Programme FP7/2007–2013 project EXPOsOMICS (grant no. 308610). M.P. was supported by the People Program (Marie Curie Actions) of the European Union's Seventh Framework Program FP7/2007–2013/ under REA grant agreement n° [628858]. A.V. has a PhD fellowship from Bijzonder Onderzoeksfonds (BOF) Hasselt University.

### Exploring Perinatal Outcomes in Children (EPOCH)

EPOCH is funded by the following NIH grants: R01DK068001; R01 DK100340.

### Flemish Environment and Health Study I (FLEHSI) birth cohort

The FLEHS study was commissioned, financed and steered by the Ministry of the Flemish Community (Department of Economics, Science and Innovation; Flemish Agency for Care and Health; and Department of Environment, Nature and Energy). The methylation work was funded by the CEFIC LRI award 2013 that was given to S.L. who is the beneficiary of the Cefic-LRI Innovative Science Award 2013 and of a post-doctoral fellowship [12L5216N; http://www.fwo.be/] provided by the Research Foundation-Flanders (FWO) and the Flemish Institute for Technological Research (VITO). P.deB. is recipient of a Bill & Melinda Gates Foundation Grand Challenges Exploration grant (OPP119403) in the field of saliva biomarker discovery.

### Genes-environments and Admixture in Latino Americans (GALA II)

The GALA II study was supported in part by grants from the Sandler Family Foundation; the American Asthma Foundation; National Institutes of Health: the National Heart, Lung and Blood Institute (HL117004); the National Institute of Environmental Health Sciences (ES24844); the National Institute on Minority Health and Health Disparities (MD006902, MD009523); the National Institute of General Medical Sciences (GM007546); the Tobacco-Related Disease Research Program (24RT-0025).

### Groningen Expertise Centrum voor Kinderen met Overgewicht (GECKO)

The GECKO Drenthe birth cohort was funded by an unrestricted grant of Hutchison Whampoa Ltd, Hong Kong, and supported by the University of Groningen, Well Baby Clinic Foundation Icare, Noordlease and Youth Health Care Drenthe. This methylation project in the GECKO Drenthe cohort was supported by the Biobanking and Biomolecular Research Infrastructure Netherlands (CP2011–19).

### Generation R Study

The Generation R Study is made possible by financial support from the Erasmus Medical Center, Rotterdam, the Erasmus University Rotterdam and the Netherlands Organization for Health Research and Development. The EWAS data were funded by a grant to V.W.J. from the Netherlands Genomics Initiative (NGI)/Netherlands Organization for Scientific Research (NWO), Netherlands Consortium for Healthy Aging (NCHA; project nr. 050–060–810) and by funds from the Genetic Laboratory of the Department of Internal Medicine, Erasmus MC. This study received funding from the European Union’s Horizon 2020 research and innovation programme (733206, LifeCycle). The Generation R EWAS data were partially funded by a grant from the National Institute of Child and Human Development (R01HD068437).V.W.J. received a grant from the Netherlands Organization for Health Research and Development (VIDI 016.136.361) and a Consolidator grant from the European Research Council (ERC-2014-CoG-648916). J.F.F. has received funding from the European Union’s Horizon 2020 research and innovation programme under grant agreement No 633595 (DynaHEALTH). M.J.B-K. has received funding from the Netherlands' Organization for Scientific Research (NWO VICI) and an Advanced grant 2015 from the European Research Council ERC. M.H.V.J. has received funding from the Netherlands' Organization for Scientific Research (NWO Spinoza Award) and Gravitation program of the Dutch Ministry of Education, Culture, and Science and the Netherlands Organization for Scientific Research (NWO grant number 024.001.003). L.D. received funding from the Lung Foundation Netherlands (no 3.2.12.089; 2012).

### Genetics of Glycemic regulation in Gestation and Growth (Gen3G)

Gen3G was supported by a Fonds de Recherche du Québec en Santé (FRQ-S) operating grant (grant #20697); a Canadian Institute of Health Reseach (CIHR) operating grant (grant #MOP 115071); a Diabète Québec grant; and a Canadian Diabetes Association operating grant (grant #OG-3–08–2622). M.F.H. has received an American Diabetes Association Pathways Accelerator Early Investigator Award (No 1–15-ACE-26). L.B .is a junior scholar from Fonds de Recherche du Québec en Santé (FRQ-S).

### Genetics of Overweight Young Adults (GOYA)

Genotyping for the GOYA Study was funded by the Wellcome Trust (grant ref: 084762MA). Generation of DNA methylation data was funded by the MRC Integrative Epidemiology Unit which is supported by the Medical Research Council (MC_UU_12013/1–9) and the University of Bristol.

### Healthy Start

Healthy Start is funded by the following NIH grants: R01 DK076648; R01ES022934; UL1 TR001082 – NIH/NCATS Colorado CTSA; P30 DK56350 – UNC Nutrition Obesity Research Center. A.P.S. has received funding from the National Institute of Environmental Health Sciences, National Institutes of Health (K99ES025817).

### Infancia y Medio Ambiente (INMA)

Main funding of the epigenetic studies in INMA were grants from Instituto de Salud Carlos III (Red INMA G03/176, CB06/02/0041), Spanish Ministry of Health (FIS-PI04/1436, FIS-PI08/1151 including FEDER funds, FIS-PI11/00610, FIS-FEDER-PI06/0867, FIS-FEDER-PI03–1615) Generalitat de Catalunya-CIRIT 1999SGR 00241, Fundació La Marató de TV3 (090430), EU Commission (261357-MeDALL: Mechanisms of the Development of ALLergy), and European Research Council (268479-BREATHE: BRain dEvelopment and Air polluTion ultrafine particles in scHool childrEn).

### Inner City Asthma Consortium (ICAC) EPIGEN Cohort

Inner City Asthma Consortium EPIGEN cohort was funded by the National Institute of Allergy and Infectious Diseases (N01-AI90052).

### Isle of Wight 1989 birth cohort (IoW F1) and 3rd Generation study (IoW F2)

The IoW F1 Birth cohort assessments have been supported by the National Institutes of Health USA (grant no. R01 HL082925 and R01 HL132321) and Asthma UK (grant no. 364). The IoW third generation cohort was funded by NIAID/NIH R01AI091905. Methylation analysis was supported in part by NIAID/NIH R01AI091905 and R01AI121226. J.W.H., W.K., S.H.A. and H.Z. have received funds from the National Institute of Health R01 AI091905 (PI: Wilfried Karmaus), R01AI121226 (MPI: Hongmei Zhang and John Holloway) and R01HL132321 (PI: Wilfried Karmaus).

### Norwegian Mother and Child Cohort Study (MoBa)

The Norwegian Mother and Child Cohort Study is supported by the Norwegian Ministry of Health and Care Services and the Ministry of Education and Research, NIH/NIEHS (contract no N01-ES-75558), NIH/NINDS (grant no.1 UO1 NS 047537–01 and grant no.2 UO1 NS 047537–06A1). MoBa1 and MoBa 2 are supported by the Intramural Research Program of the NIH, National Institute of Environmental Health Sciences (Z01-ES-49019) and Norwegian Research Council/BIOBANK (grant no 221097). The work in MoBa3 was supported in part by a Postdoctoral Fellowship grant from the Ullevål Hospitals Research Council (now under Oslo University Hospital) and travel grants from the Unger-Vetlesens foundation and the Norwegian American Womens Club, all to M.C.M.K. MoBa3 epigenomics data analyses were funded by INCA/Plan Cancer-EVA-INSERM, France, and the International Childhood Cancer Cohort Consortium (I4C), and performed by the Epigenetics Group at the International Agency for Research on Cancer (IARC, Lyon, France), A.G. was supported by the grant from INCA/Plan Cancer-EVA-INSERM (France, 2015) to Z.H. and also by the IARC Postdoctoral Fellowship, partially supported by the EC FP7 Marie Curie Actions-People-Co-funding of regional, national and international programmes (COFUND).

### Norway Facial Clefts Study (NCL)

This research was supported by the Intramural Research Program of the NIH, National Institute of Environmental Health Sciences (Z01 ES044005, ES049033, ES049032).

### Newborn Epigenetics Study (NEST)

The NEST study was funded by NIEHS grants R21ES014947 and R01ES016772 and NIDDK grant R01DK085173. C.H. and D.D.J. have received funding from the National Institute of Environmental Health Science (P30 ES025128).

### Northern Finland Birth Cohorts (NFBC) 1966

NFBC1966 received financial support from University of Oulu grant no. 65354, Oulu University Hospital grant no. 2/97, 8/97, Ministry of Health and Social Affairs grant no. 23/251/97, 160/97, 190/97, National Institute for Health and Welfare, Helsinki grant no. 54121, Regional Institute of Occupational Health, Oulu, Finland grant no. 50621, 54231. S.S. has received funding from the European Union’s Horizon 2020 research and innovation programme under grant agreement No 633595 (DynaHEALTH).

### Northern Finland Birth Cohorts (NFBC) 1986

EU QLG1-CT-2000–01643 (EUROBLCS) grant no. E51560, NorFA grant no. 731, 20056, 30167, USA / NIHH 2000 G DF682 grant no. 50945. M.R.J. has received funding from the European Union’s Horizon 2020 research and innovation programme under grant agreement No. 633595 (DynaHEALTH). M.R.J. has received funding from the Academy of Finland for this work.

### New Hampshire Birth Cohort Study (NHBCS)

The NHBCS was supported by: NIH-NIEHS P01 ES022832; US EPA grant RD83544201; NIH-NIGMS P20GM104416; and NCI R25CA134286.

### Prevention and Incidence of Asthma and Mite Allergy (PIAMA)

The PIAMA study was supported by the Netherlands Organization for Health Research and Development; the Netherlands Organization for Scientific Research; the Netherlands Asthma Fund; the Netherlands Ministry of Spatial Planning, Housing, and the Environment; and the Netherlands Ministry of Health, Welfare, and Sport. Methylation analyses were supported by MeDALL, a collaborative project supported by the European Union under the Health Cooperation Work Program of the 7th Framework program (grant agreement number 261357).

### Piccoli+

The study was approved and initially funded by the Italian National Centre for Disease Prevention and Control (CCM grant 2010) and by the Italian Ministry of Health (art 12 and 12bis Dl.gs.vo 502/92). The methylation assays were funded by the European Community's Seventh Framework Programme FP7/2007–2013 project EXPOsOMICS (grant no. 308610).

### Prediction and Prevention of Preeclampsia and Intrauterine Growth Restriction Study (PREDO)

The PREDO Study has been funded by the Academy of Finland, EraNet, EVO (a special state subsidy for health science research), University of Helsinki Research Funds, the Signe and Ane Gyllenberg foundation, the Emil Aaltonen Foundation, the Finnish Medical Foundation, the Jane and Aatos Erkko Foundation, the Novo Nordisk Foundation, the Päivikki and Sakari Sohlberg Foundation and the Sigrid Juselius Foundation granted to members of the Predo study board. Methylation assays were funded by the Academy of Finland. J.L. has received funding from the University of Helsinki and Academy of Finland.

### PRISM

R.J.W. received funding for the PRISM cohort under R01 HL095606 and R01 HL1143396.

### Project Viva

The Project Viva cohort is funded by NIH grants R01HL111108, R01NR013945, and R37 HD034568.

### The Western Australian Pregnancy Cohort (Raine) Study

We would like to acknowledge the University of Western Australia (UWA), Curtin University, the Raine Medical Research Foundation, the UWA Faculty of Medicine, Dentistry and Health Sciences, the Telethon Kids Institute, the Women’s and Infant’s Research Foundation (KEMH) and Edith Cowan University for providing funding for the core management of the Raine Study. The funding for the methylation assays was through the National Health and Medical Research Council grant 1059711. R.C.H. receives funding from the National Health and Medical Research Council (NHMRC) fellowship 1053384. P.M. is supported by funding from the Australian National Health and Medical Research Council and the United States National Institute of Health.

### Rhea Mother-Child Cohort (Rhea)

The Rhea project was financially supported by European Union (EU) grants for specific projects (EU FP6–2003-Food-3-NewGeneris; EU FP6. STREP HiWATE; EU FP7 ENV.2007.1.2.2.2. Project no. 211250 ESCAPE; EU FP7–2008-ENV-1.2.1.4 Envirogenomarkers; EU FP7-HEALTH-2009-single stage CHICOS; EU FP7 ENV.2008.1.2.1.6. Proposal no. 226285 ENRIECO; EU-FP7-HEALTH-2012 Proposal no. 308333 HELIX); MeDALL (FP7 European Union project, no. 264357); and the Greek Ministry of Health (programme of prevention of obesity and neurodevelopmental disorders in preschool children, in Heraklion district, Crete, Greece, 2011–14; ‘Rhea Plus’: Primary Prevention Program of Environmental Risk Factors for Reproductive Health, and Child Health: 2012–15). The methylation assays were funded by the European Community's Seventh Framework Programme FP7/2007–2013 project EXPOsOMICS (grant no. 308610).

### Rhode Island Child Health Study (RICHS)

RICHS was supported by the National Institutes of Health (NIH-NIMH R01MH094609, NIH-NIEHS R01ES022223, NIH-NIEHS R01ES025145).

### Study to Explore Early Development, Phase I (SEED I)

The SEED study is funded by the Centers for Disease Control and Prevention (grant nos. U10DD000180, U10DD000181, U10DD000182, U10DD000183, U10DD000184, U10DD000498) and the methylation assays were funded by Autism Speaks (grant no. 7659).

### Swedish Twin study On Prediction and Prevention of Asthma (STOPPA)

Financial support was provided by the Swedish Research Council through the Swedish Initiative for research on Microdata in the Social And Medical Sciences (SIMSAM) framework grant number 340–2013–5867, grants provided by the Stockholm County Council (ALF projects), the Strategic Research Program in Epidemiology at Karolinska Institutet, the Swedish Asthma and Allergy Association’s Research Foundation, Stiftelsen Frimurare Barnahuset Stockholm and the Swedish Heart-Lung Foundation.

## Supplementary Material

Supplementary Table 1

Supplementary Material

## References

[1] Groom A, Elliott HR, Embleton ND, Relton CL Epigenetics and child health: basic principles. Arch Dis Child 2011;96**:** 863–9. 20656732 10.1136/adc.2009.165712

[2] Burris HH, Baccarelli AA Environmental epigenetics: from novelty to scientific discipline. J Appl Toxicol 2014;34**:** 113–16. 23836446 10.1002/jat.2904 PMC3867531

[3] Bakulski KM, Fallin MD Epigenetic epidemiology: promises for public health research. Environ Mol Mutagen 2014;55**:** 171–83. 24449392 10.1002/em.21850 PMC4011487

[4] Marsit CJ Influence of environmental exposure on human epigenetic regulation. J Exp Biol 2015;218 **(**Pt 1):71–79. 25568453 10.1242/jeb.106971 PMC4286705

[5] Reik W, Dean W, Walter J Epigenetic reprogramming in mammalian development. Science 2001;293**:** 1089–93. 11498579 10.1126/science.1063443

[6] Godfrey KM, Costello PM, Lillycrop KA The developmental environment, epigenetic biomarkers and long-term health. J Dev Orig Health Dis 2015;6**:** 399–406. 26017068 10.1017/S204017441500121X PMC4789489

[7] Barker DJ Fetal origins of coronary heart disease. BMJ 1995;311**:** 171–74. 7613432 10.1136/bmj.311.6998.171 PMC2550226

[8] Gluckman PD, Hanson MA, Cooper C, Thornburg KL Effect of in utero and early-life conditions on adult health and disease. N Engl J Med 2008;359**:** 61–73. 18596274 10.1056/NEJMra0708473 PMC3923653

[9] Godfrey KM, Barker DJ Fetal nutrition and adult disease. Am J Clin Nutr 2000;71 **(****Suppl 5**):1344–52S. 10.1093/ajcn/71.5.1344s 10799412

[10] Moller SE, Ajslev TA, Andersen CS, Dalgard C, Sorensen TI Risk of childhood overweight after exposure to tobacco smoking in prenatal and early postnatal life. PLoS One 2014;9**:** e109184. 25310824 10.1371/journal.pone.0109184 PMC4195647

[11] Roseboom TJ, Painter RC, van Abeelen AF, Veenendaal MV, de Rooij SR Hungry in the womb: what are the consequences? Lessons from the Dutch famine. Maturitas 2011;70**:** 141–45. 21802226 10.1016/j.maturitas.2011.06.017

[12] Yu Z, Han S, Zhu J, Sun X, Ji C, Guo X Pre-pregnancy body mass index in relation to infant birth weight and offspring overweight/obesity: a systematic review and meta-analysis. PLoS One 2013;8**:** e61627. 23613888 10.1371/journal.pone.0061627 PMC3628788

[13] Richmond RC, Timpson NJ, Sorensen TI Exploring possible epigenetic mediation of early-life environmental exposures on adiposity and obesity development. Int J Epidemiol 2015;44**:** 1191–98. 25953782 10.1093/ije/dyv066 PMC4588870

[14] Reese SE, Zhao S, Wu MC, et al DNA methylation score as a biomarker in newborns for sustained maternal smoking during pregnancy. Environ Health Perspect 2017;125**:** 760–66. 27323799 10.1289/EHP333 PMC5381987

[15] Ladd-Acosta C, Fallin MD The role of epigenetics in genetic and environmental epidemiology. Epigenomics 2016;8**:** 271–83. 26505319 10.2217/epi.15.102

[16] Valeri L, Reese SL, Zhao S, et al Misclassified exposure in epigenetic mediation analyses. Does DNA methylation mediate effects of smoking on birthweight? Epigenomics 2017;9**:** 253–65. 28234025 10.2217/epi-2016-0145 PMC5331915

[17] Heijmans BT, Tobi EW, Stein AD, et al Persistent epigenetic differences associated with prenatal exposure to famine in humans. Proc Natl Acad Sci U S A 2008;105**:** 17046–49. 18955703 10.1073/pnas.0806560105 PMC2579375

[18] Benke KS, Nivard MG, Velders FP, et al A genome-wide association meta-analysis of preschool internalizing problems. J Am Acad Child Adolesc Psychiatry 2014;53**:** 667–76 e7. 24839885 10.1016/j.jaac.2013.12.028

[19] Bonnelykke K, Sleiman P, Nielsen K, et al A genome-wide association study identifies CDHR3 as a susceptibility locus for early childhood asthma with severe exacerbations. Nat Genet 2014;46**:** 51–55. 24241537 10.1038/ng.2830

[20] Bradfield JP, Taal HR, Timpson NJ, et al A genome-wide association meta-analysis identifies new childhood obesity loci. Nat Genet 2012;44**:** 526–31. 22484627 10.1038/ng.2247 PMC3370100

[21] Felix JF, Bradfield JP, Monnereau C, et al Genome-wide association analysis identifies three new susceptibility loci for childhood body mass index. Hum Mol Genet 2016;25**:** 389–403. 26604143 10.1093/hmg/ddv472 PMC4854022

[22] Horikoshi M, Yaghootkar H, Mook-Kanamori DO, et al New loci associated with birth weight identify genetic links between intrauterine growth and adult height and metabolism. Nat Genet 2013;45**:** 76–82. 23202124 10.1038/ng.2477 PMC3605762

[23] Pappa I, St Pourcain B, Benke K, et al A genome-wide approach to children's aggressive behavior: The EAGLE consortium. Am J Med Genet B Neuropsychiatr Genet 2016;161**:** 562–72. 10.1002/ajmg.b.32333 26087016

[24] Paternoster L, Standl M, Waage J, et al Multi-ancestry genome-wide association study of 21, 000 cases and 95, 000 controls identifies new risk loci for atopic dermatitis. Nat Genet 2015;47**:** 1449–56. 26482879 10.1038/ng.3424 PMC4753676

[25] van der Valk RJ, Duijts L, Timpson NJ, et al Fraction of exhaled nitric oxide values in childhood are associated with 17q11.2-q12 and 17q12-q21 variants. J Allergy Clin Immunol 2014;134**:** 46–55. 24315451 10.1016/j.jaci.2013.08.053 PMC4334587

[26] Bibikova M, Barnes B, Tsan C, et al High density DNA methylation array with single CpG site resolution. Genomics 2011;98**:** 288–95. 21839163 10.1016/j.ygeno.2011.07.007

[27] Moran S, Arribas C, Esteller M Validation of a DNA methylation microarray for 850, 000 CpG sites of the human genome enriched in enhancer sequences. Epigenomics 2016;8**:** 389–99. 26673039 10.2217/epi.15.114 PMC4864062

[28] Engel SM, Joubert BR, Wu MC, et al Neonatal genome-wide methylation patterns in relation to birth weight in the Norwegian Mother and Child Cohort. Am J Epidemiol 2014;179**:** 834–42. 24561991 10.1093/aje/kwt433 PMC3969535

[29] Joubert BR, Haberg SE, Nilsen RM, et al 450 K epigenome-wide scan identifies differential DNA methylation in newborns related to maternal smoking during pregnancy. Environ Health Perspect 2012;120**:** 1425–31. 22851337 10.1289/ehp.1205412 PMC3491949

[30] Richmond RC, Simpkin AJ, Woodward G, et al Prenatal exposure to maternal smoking and offspring DNA methylation across the lifecourse: findings from the Avon Longitudinal Study of Parents and Children (ALSPAC). Hum Mol Genet 2015;24**:** 2201–17. 25552657 10.1093/hmg/ddu739 PMC4380069

[31] Sharp GC, Lawlor DA, Richmond RC, et al Maternal pre-pregnancy BMI and gestational weight gain, offspring DNA methylation and later offspring adiposity: findings from the Avon Longitudinal Study of Parents and Children. Int J Epidemiol 2015;44**:** 1288–304. 25855720 10.1093/ije/dyv042 PMC4588865

[32] Joubert BR, Felix JF, Yousefi P, et al DNA methylation in newborns and maternal smoking in pregnancy: genome-wide consortium meta-analysis. Am J Hum Genet 2016;98**:** 680–96. 27040690 10.1016/j.ajhg.2016.02.019 PMC4833289

[33] Psaty BM, O'Donnell CJ, Gudnason V, et al Cohorts for Heart and Aging Research in Genomic Epidemiology (CHARGE) Consortium: Design of prospective meta-analyses of genome-wide association studies from 5 cohorts. Circ Cardiovasc Genet 2009;2**:** 73–80. 20031568 10.1161/CIRCGENETICS.108.829747 PMC2875693

[34] Willer CJ, Li Y, Abecasis GR METAL: fast and efficient meta-analysis of genomewide association scans. Bioinformatics 2010;26**:** 2190–91. 20616382 10.1093/bioinformatics/btq340 PMC2922887

[35] Gruzieva O, Xu CJ, Breton CV, et al Epigenome-wide meta-analysis of methylation in children related to prenatal NO2 air pollution exposure. Environ Health Perspect 2017;125**:** 104–10. 27448387 10.1289/EHP36 PMC5226705

[36] Lawlor DA, Harbord RM, Sterne JA, Timpson N, Davey Smith G Mendelian randomization: using genes as instruments for making causal inferences in epidemiology. Stat Med 2008;27**:** 1133–63. 17886233 10.1002/sim.3034

[37] Relton CL, Davey Smith G Epigenetic epidemiology of common complex disease: prospects for prediction, prevention, and treatment. PLoS Med 2010;7**:** e1000356. 21048988 10.1371/journal.pmed.1000356 PMC2964338

[38] Relton CL, Davey Smith G Two-step epigenetic Mendelian randomization: a strategy for establishing the causal role of epigenetic processes in pathways to disease. Int J Epidemiol 2012;41**:** 161–76. 22422451 10.1093/ije/dyr233 PMC3304531

[39] Ladd-Acosta C, Shu C, Lee BK, et al Presence of an epigenetic signature of prenatal cigarette smoke exposure in childhood. Environ Res 2016;144 **(**Pt A):139–48. 26610292 10.1016/j.envres.2015.11.014 PMC4915563

[40] Yousefi P, Huen K, Dave V, Barcellos L, Eskenazi B, Holland N Sex differences in DNA methylation assessed by 450 K BeadChip in newborns. BMC Genomics 2015;16**:** 911. 26553366 10.1186/s12864-015-2034-y PMC4640166

[41] Rijlaarsdam J, Pappa I, Walton E, et al An epigenome-wide association meta-analysis of prenatal maternal stress in neonates: A model approach for replication. Epigenetics 2016;11**:** 140–49. 26889969 10.1080/15592294.2016.1145329 PMC4846102

[42] Relton CL, Gaunt T, McArdle W, et al Data Resource Profile: Accessible Resource for Integrated Epigenomic Studies (ARIES). Int J Epidemiol 2015;44**:** 1181–90. 25991711 10.1093/ije/dyv072 PMC5593097

[43] Rzehak P, Saffery R, Reischl E, et al Maternal smoking during pregnancy and DNA methylation in children at age 5.5 years: epigenome-wide-analysis in the European Childhood Obesity Project (CHOP) Study. PLoS One 2016;11**:** e0155554. 27171005 10.1371/journal.pone.0155554 PMC4865176

[44] Joubert BR, den Dekker HT, Felix JF, et al Maternal plasma folate impacts differential DNA methylation in an epigenome-wide meta-analysis of newborns. Nat Commun 2016;7**:** 10577. 26861414 10.1038/ncomms10577 PMC4749955

[45] Kupers LK, Xu X, Jankipersadsing SA, et al DNA methylation mediates the effect of maternal smoking during pregnancy on birthweight of the offspring. Int J Epidemiol 2015;44**:** 1224–37. 25862628 10.1093/ije/dyv048 PMC4588868

[46] Everson TM, Lyons G, Zhang H, et al DNA methylation loci associated with atopy and high serum IgE: a genome-wide application of recursive random forest feature selection. Genome Med 2015;7**:** 89. 26292806 10.1186/s13073-015-0213-8 PMC4545869

[47] Lockett GA, Soto-Ramirez N, Ray MA, et al Association of season of birth with DNA methylation and allergic disease. Allergy 2016;71**:** 1314–24. 26973132 10.1111/all.12882 PMC5639882

[48] Yousefi P, Huen K, Aguilar Schall R, et al Considerations for normalization of DNA methylation data by Illumina 450 K BeadChip assay in population studies. Epigenetics 2013;8**:** 1141–52. 23959097 10.4161/epi.26037 PMC6242262

[49] Yousefi P, Huen K, Quach H, et al Estimation of blood cellular heterogeneity in newborns and children for epigenome-wide association studies. Environ Mol Mutagen 2015;56**:** 751–58. 26332589 10.1002/em.21966 PMC4636959

[50] Zhong J, Agha G, Baccarelli AA The role of DNA methylation in cardiovascular risk and disease: methodological aspects, study design, and data analysis for epidemiological studies. Circ Res 2016;118**:** 119–31. 26837743 10.1161/CIRCRESAHA.115.305206 PMC4743554

[51] Wu MC, Joubert BR, Kuan PF, et al A systematic assessment of normalization approaches for the Infinium 450 K methylation platform. Epigenetics 2014;9**:** 318–29. 24241353 10.4161/epi.27119 PMC3962542

[52] Zhao N, Bell DA, Maity A, et al Global analysis of methylation profiles from high resolution CpG data. Genet Epidemiol 2015;39**:** 53–64. 25537884 10.1002/gepi.21874 PMC4314375

[53] Oros Klein K, Grinek S, Bernatsky S, et al funtooNorm: an R package for normalization of DNA methylation data when there are multiple cell or tissue types. Bioinformatics 2016;32**:** 593–95. 26500152 10.1093/bioinformatics/btv615 PMC4743629

[54] Aryee MJ, Jaffe AE, Corrada-Bravo H, et al Minfi: a flexible and comprehensive bioconductor package for the analysis of Infinium DNA methylation microarrays. Bioinformatics 2014;30**:** 1363–69. 24478339 10.1093/bioinformatics/btu049 PMC4016708

[55] Gruzieva O, Xu CJ, Breton CV, et al Epigenome-wide meta-analysis of methylation in children related to prenatal NO2 air pollution exposure. Environ Health Perspect 2017;125**:** 104–10. 27448387 10.1289/EHP36 PMC5226705

[56] Houseman EA, Accomando WP, Koestler DC, et al DNA methylation arrays as surrogate measures of cell mixture distribution. BMC Bioinformatics 2012;13**:** 86. 22568884 10.1186/1471-2105-13-86 PMC3532182

[57] Reinius LE, Acevedo N, Joerink M, et al Differential DNA methylation in purified human blood cells: implications for cell lineage and studies on disease susceptibility. PLoS One 2012;7**:** e41361. 22848472 10.1371/journal.pone.0041361 PMC3405143

[58] Bakulski KM, Feinberg JI, Andrews SV, et al DNA methylation of cord blood cell types: Applications for mixed cell birth studies. Epigenetics. 2016;11**:** 354–62. 27019159 10.1080/15592294.2016.1161875 PMC4889293

[59] Gervin K, Page CM, Aass HC, et al Cell type specific DNA methylation in cord blood: a 450 K-reference data set and cell count-based validation of estimated cell type composition. Epigenetics 2016;11**:** 690–98. 27494297 10.1080/15592294.2016.1214782 PMC5048717

[60] Rakyan VK, Down TA, Balding DJ, Beck S Epigenome-wide association studies for common human diseases. Nat Rev Genet 2011;12**:** 529–41. 21747404 10.1038/nrg3000 PMC3508712

[61] Lehne B, Drong AW, Loh M, et al A coherent approach for analysis of the Illumina HumanMethylation450 BeadChip improves data quality and performance in epigenome-wide association studies. Genome Biol 2015;16**:** 37. 25853392 10.1186/s13059-015-0600-x PMC4365767

[62] Michels KB, Binder AM, Dedeurwaerder S, et al Recommendations for the design and analysis of epigenome-wide association studies. Nat Methods 2013;10**:** 949–55. 24076989 10.1038/nmeth.2632

[63] Xu Z, Niu L, Li L, Taylor JA ENmix: a novel background correction method for Illumina HumanMethylation450 BeadChip. Nucleic Acids Res 2016;44 **(**3):e20. 26384415 10.1093/nar/gkv907 PMC4756845

[64] Felix JF, Jaddoe VWV, Duijts L Invloed van DNA methylatie op gezondheid en ziekte van kinderen [Dutch] (Influence of DNA methylation on health and disease in children.). Kinderarts en Wetenschap 2015;16**:** 10–14.

