# Corrigendum: Cohort Profile: Pregnancy And Childhood Epigenetics (PACE) Consortium

**DOI:** 10.1093/ije/dyx220

**Published:** 2017-10-11

First published online: 13 September 2017, *Int J Epidemiol*, doi: https://doi.org/10.1093/ije/dyx190

Please note that Peter Rzehak is from the Division of Metabolic and Nutritional Medicine, Dr. von Hauner Children’s Hospital, Ludwig-Maximilians Universität München (LMU), Munich, Germany, and not the Department of Environmental Health Sciences, Columbia University Mailman School of Public Health, New York, NY, USA, as originally published online.

