# Supplementary material for: Cohort Profile: Pregnancy And Childhood Epigenetics (PACE) Consortium
Source: Int J Epidemiol. 2017 Sep 13;47(1):22–23u. doi: 10.1093/ije/dyx190 (PMC5837319; doi:10.1093/ije/dyx190)
Supplement: Supplementary Material [file dyx190_ije-2016-09-1043-file008.docx]

**Cohort Profile:**

**Pregnancy And Childhood Epigenetics (PACE) Consortium**

**SUPPLEMENTARY MATERIAL**

**Study-specific methods**

***Avon Longitudinal Study of Parents And Children (ALSPAC)***

ALSPAC is a large, prospective cohort study based in the South West of England. 14 541 pregnant women resident in Avon, UK with expected dates of delivery 1st April 1991 to 31st December 1992 were recruited and detailed information has been collected on these women and their offspring at regular intervals (1, 2). The study website contains details of all the data that is available through a fully searchable data dictionary (http://www.bris.ac.uk/alspac/researchers/data-access/data-dictionary/).

As part of the ARIES (Accessible Resource for Integrated Epigenomic Studies, http://www.ariesepigenomics.org.uk/) project, the Illumina Infinium HumanMethylation450 BeadChip (Illumina Inc., San Diego, USA) has been used to generate epigenetic data on 1018 mother-offspring pairs in the ALSPAC cohort (3, 4). The ARIES participants were selected based on availability of DNA samples at two time points for the mother (antenatal and at follow-up when the offspring were adolescents) and three time points for the offspring (neonatal, childhood (age 7) and adolescence (age 17)). Written informed consent has been obtained for all ALSPAC participants. Ethical approval for the study was obtained from the ALSPAC Ethics and Law Committee and the Local Research Ethics Committees.

Cord blood and peripheral blood samples (whole blood, buffy coats or blood spots) were collected according to standard procedures. The DNA methylation wet-lab and pre-processing analyses were performed at the University of Bristol as part of the ARIES project. Following extraction, DNA was bisulphite-converted using the Zymo EZ DNA MethylationTM kit (Zymo, Irvine, CA, USA). Following conversion, genome-wide methylation status of over 485 000 CpG sites was measured using the Illumina Infinium HumanMethylation450 BeadChip according to the standard protocol. The arrays were scanned using an Illumina iScan and initial quality review was assessed using GenomeStudio (version 2011.1). Samples from all time points in ARIES were distributed across slides using a semi-random approach (sampling criteria were in place to ensure that all time points were represented on each array) to minimise the possibility of confounding by batch effects. In addition, during the data generation process, a wide range of batch variables were recorded in a purpose-built laboratory information management system (LIMS). The main batch variable was found to be the bisulphite conversion (BCD) plate number. Samples were converted in batches of 48 samples and each batch identified by a plate number. The LIMS also reported quality control (QC) metrics from the standard control probes on the Illumina Infinium HumanMethylation450 BeadChip for each sample. Samples failing QC (average probe *P*-value >= 0.01) were repeated and if unsuccessful excluded from further analysis. As an additional QC step genotype probes were compared with SNP-chip data from the same individual to identify and remove any sample mismatches. For individuals with no genome-wide SNP data, samples were flagged if there was a sex-mismatch based on X-chromosome methylation.

***Children’s Allergy Environment Stockholm Epidemiology study (BAMSE)***

BAMSE is a prospective population-based cohort study of children recruited at birth and followed during childhood and adolescence. Details of the study design, inclusion criteria, enrollment and data collection are described elsewhere (5). In short, 4089 children born between 1994 and 1996 in four municipalities of Stockholm County, Sweden were enrolled. At baseline, when the infant was approximately 2 months of age, parents completed a questionnaire that assessed residential characteristics, as well as socioeconomic and lifestyle factors, including parental smoking habits. When children were 1, 2, 4, 8, 12 and 16 years, the parents completed questionnaires focusing on children’s symptoms related to wheezing and allergic diseases, as well as various exposures. The survey response rates were 96%, 94%, 91%, 84%, 82% and 78%, respectively. Furthermore, blood was obtained from 2614 (64%), 2480 (61%), and 2547 (62%) of the children at the age of 4, 8 and 16 years, respectively. The baseline and follow-up studies were approved by the Regional Ethical Review Board, Karolinska Institutet, Stockholm, Sweden, and the parents of all participating children provided informed consent.

Epigenome-wide DNA methylation was measured in 472 Caucasian children, using DNA

extracted from blood samples collected at the age of 8 years (BAMSE EpiGene). 500 ng DNA per sample

underwent bisulfite conversion using the EZ-96 DNA Methylation kit (Shallow) (Zymo Research

Corporation, Irvine, USA). Samples were plated onto 96-well plates in randomized order. Samples were processed with the Illumina Infinium HumanMethylation450 BeadChip (Illumina Inc., San Diego, USA) (3). Quality control of analyzed samples was performed using standardized criteria. Samples were excluded in case of sample call rate <99%, colour balance >3, low staining efficiency, poor extension efficiency, poor hybridization performance, low stripping efficiency after extension and poor bisulfite conversion. We also applied multidimensional scaling (MDS) plot to evaluate gender outliers based on chromosome X data that produced two separated clusters for male and female. We omitted five samples that do not belong to the distinct cluster. Furthermore, we applied median intensity plot for methylated and unmethylated intensity by using the minfi R package (three samples below the 10.5 cutoff were excluded). All criteria described above led to exclusion of 8 samples. Probes with a single nucleotide polymorphism in the single base extension site with a frequency of > 5% were excluded (6), as were probes with non-optimal binding (non-mapping or mapping multiple times to either the normal or the bisulphite-converted genome), and the probes belonging to chromosome X and chromosome Y, resulting in the exclusion of 46 799 probes, leaving a total of 438 713 probes in the analysis. Furthermore, we implemented “DASEN” recommended from watermelon package to do signal correction and normalization (7). In addition, 278 paired samples collected at 4 and 8 years were analyzed in the framework the MeDALL (Mechanisms of the Development of ALLergy) collaborative project supported by the European Union under the Health Cooperation Work Programme of the 7th Framework programme (grant agreement number 261357) (8). DNA methylation measurements with Illumina Infinium HumanMethylation450 BeadChip and quality control of these samples were performed following the same procedures as described above for BAMSE EpiGene.

***California Birth Cohort (CBC)***
The California Department of Public Health maintains a repository of neonatal birth bloods as blood dried on a filter paper (Guthrie card). These are available for qualified researchers to perform specified health research as monitored by local and State level institutional review boards. Our current research project using CBC resources is a case-control study of childhood leukemia, the California Childhood Leukemia Study (CCLS) which identifies children with leukemia and matched controls (birthdate, gender, and ethnicity) from around the State of California (9). Parents were interviewed to provide data on pre-pregnancy, pregnancy, and early life exposures in the topics of diet, smoking, radiation, pesticides, infections, and other variables of interest for cancer exposure. Parents provided consent to obtain birth bloods, which were used for DNA methylation analyses.

Approximately 300-500 ng of high molecular weight DNA was extracted from a ¼ section of a 1.5 cm^2^ archived neonatal DBS (stored at -20°C from the time of birth) using Qiagen blood card extraction protocol and bisulfite treated using the EZ DNA Methylation-Direct™ Kit (Zymo). Genome-wide DNA methylation was then measured in these bisulfite converted DNA samples using Illumina Infinium HumanMethylation450 BeadChip arrays (Illumina Inc., San Diego, USA) (3). CpG sites with detection *P*-values > 0.01 were defined as bad CpG sites and discarded. CpG sites with >15% of absence of information (i.e. >15% of total samples) were excluded. A total of 540 CpGs were excluded. Samples with >15% of bad CpG sites (of the 450K loci) were also excluded from the analysis. The DNA methylation data preprocessing consisted of functional normalization according to Fortin *et al* to control for batch and position effects (10). Additional correction for probe types was accomplished with BMIQ normalization (11).

***Center for Health Assessment of Mothers and Children of Salinas (CHAMACOS)***

The CHAMACOS study is a longitudinal birth cohort study of the effects of exposure to pesticides and environmental chemicals on the health and development of Mexican-American children living in the agricultural region of Salinas Valley, CA. Detailed description of the CHAMACOS cohort has previously been published (12, 13). Briefly, 601 pregnant women were enrolled in 1999-2000 at community clinics and 527 liveborn singletons were born and followed to delivery. Follow up visits occurred at regular intervals throughout childhood. Study protocols were approved by the University of California, Berkeley Committee for Protection of Human Subjects and written informed consent was obtained from all mothers.

DNA methylation was measured in DNA isolated from the cord blood of 378 CHAMACOS newborns by Illumina Infinium HumanMethylation450 BeadChip (Illumina Inc., San Diego, USA)(3). DNA samples were bisulfite converted using Zymo Bisulfite Conversion Kits (Zymo Research, Irvine, CA), whole genome amplified, enzymatically fragmented, purified, and applied to the Illumina Infinium HumanMethylation450 BeadChip according to manufacturer protocol. 450K BeadChips were handled by robotics and analyzed using the Illumina Hi-Scan system. DNA methylation was measured at 485 512 CpG sites. Probe signal intensities were extracted by Illumina GenomeStudio software (version XXV2011.1, Methylation Module 1.9) methylation module and background subtracted. QA/QC was performed systematically by assessment of assay repeatability batch effects using 38 technical replicates, and data quality established as previously described (14). Quality was also ensured by only retaining samples where 95% of sites assayed had detection *P*-values > 0.01. The same threshold (95% detection at *P*-value>0.01) was imposed to CpGs as well (n= 460 removed). Sites with annotated probe SNPs and with common SNPs (minor allele frequency >5%) within 50bp of the target identified in the MXL (Mexican ancestry in Los Angeles, California) HapMap population were excluded from analysis (n=49 748). This left a total of 435 369 CpGs in the analysis. In the secondary model, color channel bias, batch effects and difference in Infinium chemistry were minimized by application of ASMN algorithm (14), followed by BMIQ normalization (11). Data on effects of cell composition in cord blood and on sex differences in DNA methylation in CHAMACOS newborns have been recently published (15, 16). CHAMACOS cohort study also has Illumina Infinium HumanMethylation450 BeadChip for the subset of children at several time points (2, 5, 9 and 12 years).

***Childhood Obesity Project (CHOP)***

The CHOP study is an ongoing European multicenter randomized prospective nutritional intervention study in 1678 healthy term newborns recruited between October 1, 2002 and July 31, 2004. Currently, infants are followed up until the age of 11 years. Main objective of this study is to assess the effect of early and later nutrition on children’s weight development, growth, body composition and risk of obesity and the role epigenetic and metabolic programming plays in this context. A detailed description of the study design and the comprehensive prospective measurements can be found in recent publications (17-20). The local ethics committees of each study center approved all study procedures: Belgium (Comitè d’Ethique de L’Hopital Universitaire des Enfants Reine Fabiola; no. CEH 14/02), Germany (Bayerische Landesärztekammer Ethik-Kommission; no. 02070), Italy (Azienda Ospedaliera San Paolo Comitato Etico; no. 14/2002), Poland (Instytut Pomnik–Centrum Zdrowia Dziecka Komitet Etyczny; no 243/KE/2001), and Spain (Comité ético de investigación clinica del Hospital Universitario de Tarragona Joan XXIII). Written informed parental consent was obtained for each participating infant and from children of age 8 years onwards.

Epigenome-wide DNA methylation was measured with the Illumina Infinium HumanMethylation450 BeadChip (Illumina Inc., San Diego, USA) in 384 children of age 5.5 years (3). Briefly, genomic DNA was extracted from peripheral blood cells from buffy coats, bisulfite converted (800 ng) with the EZ-96 DNA Methylation Kit (Zymo Research, Irvine, Ca; USA) and finally hybridised on the Illumina Infinium HumanMethylation450 BeadChip arrays at the Genome Analysis Center of Helmholtz Zentrum Muenchen, Munich, Germany. Details on pre-processing, normalization and quality control were previously described (19). In brief, raw methylation data were pre-processed and normalized according to the approach of Touleimat and Tost with the modification of a beta-mixture quantile normalization (BMIQ) step (11, 21). Quality control was conducted according to standard criteria: Retaining only probes with signals from ≥3 beads, detection *P*-values≤0.01 and samples with ≥80% significant probe methylation signals per sample. In addition color bias correction and background adjustment were conducted with R-package lumi. However, except for identified cross-binding probes (6), no probe filtering according to proximity of CpG site with SNPs of minor allele frequency ≥5% within 50bp or probes on the X and Y chromosomes were conducted. In total, 431 313 CpG methylation values in each of 384 children of age 5.5 years were available for EWAS analysis before potential trimming of calculated beta-values.

***Children’s Health Study (CHS)***

The Children’s Health Study (CHS) is a population-based prospective cohort study from age 5 onwards in Southern California, which has been described in detail elsewhere (22). The study protocol was approved by the University of Southern California Institutional Review Board and informed, written consent and assent were provided by the parents and children respectively. A total of 5341 children were recruited, all of whom were born between 1995 and 1997 and are currently being followed until age 18.

Epigenome-wide DNA methylation was measured in 273 Hispanic and non-Hispanic white children, using DNA extracted from newborn bloodspots archived by the state of California. Laboratory personnel performing DNA methylation analysis were blinded to study subject information. DNA was extracted from whole blood cells using the QiaAmp DNA blood kit (Qiagen Inc, Valencia, CA) and stored at -80 degrees Celcius. 700-1000ng of genomic DNA from each sample was treated with bisulfite using the EZ-96 DNA Methylation Kit™ (Zymo Research, Irvine, CA, USA), according to the manufacturer’s recommended protocol and eluted in 18 ul. The results of Illumina Infinium HumanMethylation450 BeadChip (Illumina Inc., San Diego, USA) were compiled for each locus as previously described and were reported as beta (β) values (3, 23). Quality control of analyzed samples was performed using standardized criteria. CpG loci on the Illumina Infinium HumanMethylation450 BeadChip were removed from analyses if they were on the X and Y chromosomes, or if they contained SNPs, deletions, repeats, or if they have more than 10% missing values, leaving a total of 384 310 probes in the analysis. Illumina Infinium HumanMethylation450 BeadChip data were processed in the minfi package (version 1.16.0) in R (24), after which a normal-exponential background correction with dye bias correction was applied to the raw intensities at the array level to reduce background noise (25). We then normalized each sample’s methylation values to have the same quantiles to address sample to sample variability (21). Beta-values were calculated for all CpG sites. Beta values were considered as outliers and were removed if they fall below Quartile 1-3×IQR or above Quartile 3+3×IQR.

***Early Autism Risk Longitudinal Investigation cohort (EARLI)***

EARLI is an enriched risk prospective birth cohort to study autism etiology (26). The EARLI study was reviewed and approved by Human Subjects Institutional Review Boards (IRBs) from each of the four study sites (Johns Hopkins University, Drexel University, University of California Davis, and Kaiser Permanente Northern California). This longitudinal study recruited mothers of confirmed ASD children who were early in a subsequent pregnancy or were trying to become pregnant. There were 232 mothers with a subsequent sibling born through this study. All children were born between November 2009 and March 2012. Demographics, maternal behaviors, food frequency, medical history were all collected via questionnaire. Biosamples and house samples were collected during pregnancy, at birth, and during development. Measures of environmental exposures during pregnancy including metals, air pollution, PBDEs, and one-carbon metabolites are available and ongoing. Children participated in extensive neurophenotyping from 6 months of age until 3 years. All family members have genome-wide genotype information available and gene expression data is available on a subset of participants.

Biospecimens including cord blood and placenta were collected and archived at 213 births. Cord blood DNA was extracted using the DNA Midi kit (Qiagen, Valencia, CA) and samples were bisulfite treated and cleaned using the EZ DNA methylation gold kit (Zymo Research, Irvine, CA). DNA was plated randomly and assayed on the Illumina Infinium HumanMethylation450 BeadChip (Illumina Inc., San Diego, USA) at the Johns Hopkins SNP Center, a shared lab and informatics operation with the Center for Inherited Disease Research (Johns Hopkins University)(3). Methylation control gradients and between-plate repeated tissue controls were used. We used the minfi library (version 1.18.2) in R (version 3.3) to process raw Illumina image files into noob background corrected methylation values (24, 25). Probes with failed detection *P*-value (>0.05) in >10% of samples were removed (n=508). Samples with discordant methylation predicted sex and observed sex were removed (n=2) as were samples that appeared as outliers on the first principal component of methylation data across the genome prior to normalization (n=2). We adjusted normalized data for batch effects using ComBat in the sva package (version 3.9.1) (27). More information about the EARLI study can be found here: <http://www.earlistudy.org/>

***Etudes des Déterminants pré et postnatals précoces du développement et de la santé de l’Enfant (EDEN)***

The EDEN study is a prospective Birth Cohort Study (https://eden.vjf.inserm.fr/), which has been described in detail elsewhere (28). Pregnant women seen for a prenatal visit at the departments of Obstetrics and Gynecology of the University Hospital of Nancy and Poitiers before their twenty-fourth week of amenorrhea were invited to participate. Enrolment started in February 2003 in Poitiers and September 2003 in Nancy; it lasted 27 months in each centre. Among eligible women, 55% (2002 women) accepted to participate. The study has been approved by the ethical committees « Comité Consultatif pour la Protection des Personnes dans la Recherche Biomédicale », Le Kremlin-Bicêtre University hospital, and « Commission Nationale de l’Informatique et des Libertés ».

DNA has been extracted from 1367 cord blood samples and 836 blood samples in 5–6-year-old children; 682 children had DNA both from cord blood and at 5–6 years. Amplified and genomic DNA samples are now stored in 96-well plates at -80°C. More than 40 single nucleotide polymorphisms (SNPs) have been genotyped either from genomic or from amplified DNA. The samples underwent bisulfite treatment using the EZ-96 DNA Methylation kit (Zymo Research Corporation, Irvine, USA), and were subsequently processed with the Illumina Infinium HumanMethylation450 BeadChip (Illumina Inc., San Diego, USA)(3). In total, 439 306 CpGs are available in children with DNA measurements.

***ENVIRonAGE***

The ENVIRonAGE cohort includes 1210 mother-infant pairs recruited since 2010, and recruitment is still ongoing. Data include mothers’ lifestyle and socio-economic status, gestational history, as well as measurements of the newborns’ blood pressure (all healthy with gestational age 37–42 weeks), toxic metals in cord blood and placenta, and *in utero* and early life exposure to fine particulates and NO_2_ using a spatial temporal interpolation method. The biobanked specimen encompass placental tissue and cord blood (whole blood and buffy coats, frozen at birth at -80**°**C), including RNA/DNA. Ethical approval was obtained by the Ethical Committee of Hasselt University and the East-Limburg Hospital.

A set of 200 children from the ENVIRonAGE-cohort is included in the EXPOsOMICS Children Studies, for which data on cord blood DNA methylation is available. Aliquots of cord blood samples (collected and frozen at birth at -80**°**C) were shipped on dry ice to the Epigenetics Group at the International Agency for Research on Cancer (IARC), Lyon, France, where DNA was extracted (QIAamp 96 DNA Blood Kit, Qiagen 51161), quantified (Quant-iT PicoGreen dsDNA Assay Kit, Molecular Probes P7589) and bisulfite converted (600 ng of DNA using EZ-96 DNA Methylation kit, Zymo Research D5004). Samples were completely randomized, and DNA methylation was measured at 485 577 CpGs using Illumina Infinium HumanMethylation450 BeadChip (Illumina Inc., San Diego, USA) (3). Raw intensity (.idat) files were handled in R using the *minfi* package to calculate the methylation level at each CpG as the beta-value (β=intensity of the methylated allele (M)/(intensity of the unmethylated allele (U) + intensity of the methylated allele (M) + 100)), and the data were exported for quality control and processing. Methylation features were filtered from cross-reactive probes and low-quality probes (probes having bead counts < 3 in at least 5% of samples). Data quality was further assessed using box plots for the distribution of methylated and unmethylated signals, and multidimensional scaling plots and unsupervised clustering were used to check for sample outliers and potential gender mismatches. Samples having >1% of CpG sites with a detection *P*-value > 0.05 were removed. After background correction and color-bias adjustment, type I and type II probe distributions were aligned using the intra-sample BMIQ normalization (11). To correct for technical confounding, chip number (Sentrix_ID) and sample position on the chip (Sentrix_Position) were included in every regression model. After quality control, the sample size of processed ENVIRonAGE samples remained 200.

***Exploring Perinatal Outcomes in Children (EPOCH)***

EPOCH is a historical prospective cohort that recruited a cohort of 604 children age 6-13 years in 2005-1010 who were offspring of singleton pregnancies and were exposed (N=100) and not exposed (N=504) to diabetes *in utero*. The mother-child pairs were members of the Kaiser Permanente of Colorado at birth (B) and at EPOCH research visit (T1). Overall, 57% of the eligible mother–child pairs participated in the study. Between 2012 and 2016 a second research visit was conducted when participating children were 12-19 years old (T2) on a total 455 (81 exposed and 374 unexposed) youth. The main goal of EPOCH is to explore the long term effects on adiposity and cardio—metabolic health conferred by *in utero* exposure to overnutrition resulted from maternal diabetes and obesity.

Genomic DNA samples were collected at T1 and methylation profiles were assessed using the Illumina Human Methylation 450K platform on peripheral blood from 81 GDM exposed and 90 unexposed children. The arrays were processed in the University of Colorado Genomics Core lab.

***Flemish Environment and Health Study I (FLEHSI) birth cohort***

The Flemish Environment and Health Study (FLEHS; 2002–2006) was established for human biomonitoring in different geographically representative samples of the Flemish population in Belgium. This biomonitoring program collects data on chemical exposures and their early biological effects in human samples, it collects individual data on lifestyle, health and environment and stores the samples in a biobank for future investigations (29). The program studies exposure trends of regulated toxic chemicals and serves also as an early warning instrument for upcoming new chemicals that are introduced in the environment and to which humans are exposed. Within the FLEHSI campaign we recruited mother-newborn pairs to monitor early-life exposures, adolescents of 14–15 years, and adults (50-65 years) to monitor exposures that are more specific for these periods in life. The FLEHSI birth cohort consist of 1196 mother–child pairs who were systematically recruited between September 2002 and February 2004 via 25 maternities across Flanders. Cord blood samples were collected at delivery. Details of the recruitment protocol have been previously reported (30). Inclusion criteria were living for at least five years in the area of interest and being able to fill out Dutch questionnaires. Informed consent was provided by all participating mothers and the campaign was approved by the ethical committee of the University of Antwerp. During follow-up of the cohort at 10 years of age (n=595) data on growth, (sexual) development, diet, physical activity, medical conditions and life-style were gathered. A subcohort of those followed-up at age 10 years agreed to provide blood (n=99) and saliva (n=133) samples plus questionnaire data at the age of 11 years.

Peripheral blood mononuclear cells (PBMC) were isolated from the cord blood and blood collected at age 11years using Lymphoprep™ (Axis-Shield, Oslo, Norway). Whole genomic DNA was isolated from the cord blood PBMC fraction, and from the blood PBMC fraction and the saliva collected at age 11 years. About 500 ng of gDNA was bisulfite converted using the EZ DNA methylation kit (Zymo Research, Cambridge Bioscience, Cambridge, UK) according to manufacturer’s instructions. Genome-wide DNA methylation profiles were generated with Infinium HumanMethylation450 BeadChip Array (Illumina, San Diego, CA, USA) according to the standard Infinium HD Assay Methylation Protocol Guide (Part #15019519, Illumina). The BeadChip images were captured using the Illumina iScan. The raw methylation intensities for each probe were represented as methylation β-values (ranging from 0, unmethylated, to 1, fully methylated) and extracted from GenomeStudio Methylation Module software without background correction and normalization. Data were analyzed using the different R-packages on the freely available statistical software platform R. Raw data analysis, QC and normalization were performed using “minfi” R-package. In brief, the raw Red/Green channel data from the 450K-llumina methylation array were read by the ‘read.450k.exp’ function, converted to methylation values by ‘preprocessRaw’ and subsequently normalized using ‘preprocessSWAN’, an implementation of the Subset-quantile Within Array Normalization (SWAN) normalization procedure (31). Principal component analysis and unsupervised clustering were used to check for sample outliers and gender mismatches were removed. All samples passed quality controls and were loaded into the IMA-package used for further processing. Samples having >75% of CpG sites with a detection *P*-value > 1e-^05^ were removed (all samples passed this filer). Probes with a detection *P*-value greater than 0.01 in all samples and on the X and Y chromosome were removed. To estimate the proportion of various cell types in saliva and PBMC samples the statistical deconvolution method described by Houseman and colleagues and implemented in minfi-package as the ‘estimateCellCountsMset’ function was used (32). Reference methylomes from leukocyte subtypes were obtained from the study of Reinius et al. (33). Buccal epithelial cells reference methylomes were obtained from the GEO dataset GSE48472 (34).

***Genes-environments and Admixture in Latino Americans (GALA II)***

The Genes-environments & Admixture in Latino Americans (GALA II) study is a case-control study initiated in 2008 designed to investigate genetic, behavioral, social, and environmental determinants of asthma risk and morbidity among children aged 8-21 years, as previously described in detail (35-37). The study used identical protocols to recruit nearly 5000 Latinos (age 8-21) from 5 recruitment centers across the US (San Francisco Bay area; Houston, TX; Chicago, IL; New York, NY; and Puerto Rico). The study was approved by each of the five sites’ institutional review boards, and all subjects provided informed consent/assent.

After examining DNA from 576 subjects for complete bisulfite conversion of DNA (Zymo Research, Irvine, CA), we randomized the samples onto the Illumina Infinium HumanMethylation450 BeadChip (Illumina Inc., San Diego, USA) (3). Raw genome-wide methylation data were loaded in the R package minfi and assessed for basic quality control metrics, including determination of poorly performing probes with insignificant detection p values above background control probes (i.e., detection *P*-value >0.01). Probes with a single nucleotide polymorphism in the single base extension site were excluded. Since our study population included both males and females, we also removed the X and Y chromosomes from the raw methylation values. A total of 321 509 methylation loci were included for analysis. We corrected for batch (microarray chip) effect using the ComBat function in the R package SVA (surrogate variable analysis) and performed SWAN normalization to correct for intra-array differences between Illumina Type I and Type II probes (31, 38). A total of 569 samples passed quality control metrics and were included in the analysis.

***Groningen Expertise Centrum voor Kinderen met Overgewicht (GECKO)***

The GECKO Drenthe cohort is a population-based birth cohort in Drenthe, a northern province in the Netherlands (39). All mothers of babies born between April 2006 and April 2007 were invited to participate during the third trimester of pregnancy. Of all 4778 infants born in this period, a total of 2874 children (60%) participated in the study and are followed until adulthood. This study has been approved by the Medical Ethical Committee of the University Medical Center Groningen and parents of all participants gave written informed consent.

From 258 infants, we used DNA which was extracted from cord blood for the epigenome-wide DNA methylation analyses. To limit batch effects, we randomized all samples on gender and smoking status. Samples (500 ng per sample) were placed on three 96-well plates. Bisulfite conversion was performed using the EZ-96 DNA methylation kit (Zymo research Corporation, Irvine, USA). Then, we processed the samples with the Infinium HumanMethylation450 BeadChip (Illumina Inc., San Diego, USA) (3). We used minfi to calculate betas and p values for all 485 577 CpGs. During the quality control, we excluded two males that clustered in the female group, based on X chromosome betas. We performed Illumina-suggested background normalization, colour correction and Subset-quantile Within Array Normalization (SWAN). We excluded one sample because it did not meet the criteria of ≥99% of the CpGs with detection *P*-value<0.05. We excluded control probes, probes on X or Y chromosomes and probes that did not meet our criteria of a detection *P*-value of <0.05 in ≥99% of the samples, resulting in 465 891 remaining CpGs.

***Generation R***

The Generation R Study is a population-based prospective cohort study designed to study early environmental and genetic determinants of growth, development and health from fetal life until young adulthood, and has been described previously in detail (40). All children were born between April 2002 and January 2006. Data collection in children and their parents include questionnaires, interviews, detailed physical and ultrasound examinations, behavioural observations, Magnetic Resonance Imaging and biological samples. The study has been approved by the Medical Ethics Committee of the Erasmus Medical Center, Rotterdam. Written informed consent was obtained for all participants.

DNA extracted (using the salting-out method) from cord blood from 979 children of European ancestry was used for the EWAS analysis. 500 ng DNA per sample underwent bisulfite conversion using the EZ-96 DNA Methylation kit (Shallow) (Zymo Research Corporation, Irvine, USA). Samples were plated onto 96-well plates in no specific order. Samples were processed with the Illumina Infinium HumanMethylation450 BeadChip (Illumina Inc., San Diego, USA) (3). Quality control of analysed samples was done using standardised criteria. Samples were excluded in case of low sample call rate (<99%, 6 samples excluded), colour balance >3 (no samples excluded), low staining efficiency (no samples excluded), poor extension efficiency (no samples excluded), poor hybridization performance (no samples excluded), low stripping efficiency after extension (no samples excluded) and poor bisulfite conversion (1 sample removed). In addition, 2 samples were excluded because of a sex mismatch and 1 sample was excluded because of a retracted informed consent, leaving a total of 969 Generation R samples available for EWAS. A further 500 samples at birth, and at ages 6 and 9 years will be available in the near future. More information about the Generation R Study can be found on: [www.generationr.nl](http://www.generationr.nl).

***Genetics of Glycemic regulation in Gestation and Growth (Gen3G)***

Gen3G is a prospective observational cohort study aiming to increase our understanding of biological, environmental, and genetic determinants of glucose regulation during pregnancy and their impact on fetal development and was described in details previously (41). In brief, we recruited a total of 1034 pregnant women between January 2010 and June 2013 representing the general population of women in reproductive age receiving care at our institution. The study protocol was approved by the Centre Hospitalier Universitaire de Sherbrooke (CHUS) ethic committee board and every participant gave written informed consent before enrolment in the study, in accordance with the Declaration of Helsinki. During the first research visit (between 5 and 16 weeks of gestation), we collected demographic data, medical history, and anthropometric measurements; women completed questionnaires about lifestyle, and we collected extra blood samples during the clinically indicated blood draw. Women were excluded if they had non-singleton pregnancy, known pre-pregnancy diabetes or overt diabetes diagnosed based on biochemical screening that we performed at first trimester. During the second visit (between 24 and 30 weeks), we updated medical history, repeated anthropometry measures, and women completed the same questionnaires about lifestyle. We collected extra blood samples at each point of the clinically indicated 75g oral glucose tolerance test (fasting, 1h, and 2h). At the end of pregnancy, we collected clinical data from electronic hospital records and successfully collected cord blood and/or placenta samples in 736 deliveries. Gen3G is currently following up families 3 and 5 years following delivery, collecting updates on health of mothers and children, and multiples phenotypes related to anthropometry, metabolic outcomes, and neurodevelopment, in addition to additional bio-samples.

Among our overall population, we randomly selected 182 mother-child dyads with complete maternal and neonatal data and bio-samples, including cord blood and placenta samples. DNA samples were isolated using the Gentra Puregene Blood Kit (Qiagen, Mississauga, ON, Canada). DNA was quantified on a Beckman Coulter DTX 880 spectrophotometer using the Quant-iT™ PicoGreen® dsDNA assay kit (Life Technologies (Invitrogen), Burlington, ON, Canada) following the manufacturer’s standard procedure for a high-range standard curve. We used HumanMethylation450 BeadChips (Illumina, Inc., San Diego, CA, USA) to measure DNAm levels across the genome. We removed outliers (based on multidimensional scaling plot), sex mismatch, and samples with more than 5% of missing values across the epigenome (detection *P*- value > 0.01). After quality control, DNAm levels from HumanMethylation450 BeadChips were available in 172 placenta and 176 cord blood samples.

***Genetics of Overweight Young Adults (GOYA)***

The Genetics of Overweight Young Adults (GOYA) study is described in (42). It includes a subset of 91 387 pregnant women recruited to the Danish National Birth Cohort during 1996–2002. Of 67 853 women who had given birth to a live born infant, had provided a blood sample during pregnancy and had BMI information available, 3.6% of these women with the largest residuals from the regression of BMI on age and parity (all entered as continuous variables) were selected for GOYA. The BMI for these 2451 women ranged from 32.6 to 64.4. From the remaining cohort, a random sample of similar size (2450) was also selected. In total, 3908 mothers were successfully genotyped. DNA methylation data were generated for the offspring of 1000 mothers in the GOYA study, equally distributed between “cases” with a BMI>32 and “controls” who were sampled from the remaining BMI distribution. Phenotype data was collected during four telephone interviews, two interviews in pregnancy at approximately 16 and 30 weeks’ gestation and two interviews at six months and 18 months postpartum. Further Information about the child (for example, childhood BMI, asthma) was obtained from a questionnaire seven years after the birth. Register linkage was used to identify diseases treated in hospital.

Cord blood was collected according to standard procedures, spun and frozen at -80˚C. DNA methylation analysis and data pre-processing were performed at the University of Bristol. Following extraction, DNA was bisulfite converted using the Zymo EZ DNA MethylationTM kit (Zymo, Irvine, CA). Following conversion, the genome-wide methylation status of over 485 000 CpG sites was measured using the Illumina Infinium® HumanMethylation450k BeadChip assay according to the standard protocol. The arrays were scanned using an Illumina iScan and initial quality review was assessed using GenomeStudio (version 2011.1). The level of methylation is expressed as a “Beta” value (β-value), ranging from 0 (no cytosine methylation) to 1 (complete cytosine methylation). Samples failing quality control (average probe detection *P*-value ≥ 0.01) were repeated. Data were normalized using the functional normalization approach in the Minfi R package.

***Healthy Start***

The Healthy Start study is an ongoing pre-birth cohort in Colorado, aiming to investigate the developmental overnutrition pathway that links *in utero* metabolic and lifestyle exposures to neonatal and early childhood growth, body composition, adiposity and cardio-metabolic outcomes. The cohort was initially funded in July 2009 for a period of 5 years (Healthy Start 1 -HS1). Recruitment of pregnant women has been completed from the obstetric clinics at the University of Colorado Hospital Outpatient Pavilion. Eligible women were 16 years and older, no prior stillbirths, with singleton births, and a gestational age < 24 weeks. We have successfully recruited 1410 pregnant women, approximately 50% of the eligible pool; recruitment ended in May 2014. Participating mothers were followed until delivery and, with support from the Colorado Clinical & Translational Sciences Institute, infants were followed until approximately 24 months of age. Healthy Start 2 (HS2) was funded in August 2014 to follow offspring (and their mothers) through ages 4-5 years with important expansion of collected postnatal information. An ancillary study was also funded to add selected endocrine disrupting chemicals (EDCs) in maternal samples and epigenetics in cord blood. We conducted three in-person pregnancy visits: P1 (< 23 weeks of gestation), P2 (24-28 weeks), P3 (delivery), and two early life postnatal offspring evaluations: PN1 (4-6 months) and PN2 (18-24 months) (Healthy Start 1). We are currently examining youth for PN3 at age 4-5 years (Healthy Start 2, 2014-2019). Cord blood was drawn at the time of delivery.

Epigenetic profiling of cord blood samples (N=600) was accomplished using the Illumina 450k methylation array platform. The arrays were processed in the University of Colorado Genomics Core lab.

***Infancia y Medio Ambiente (INMA)***

The INMA—INfancia y Medio Ambiente—(Environment and Childhood) Project is a network of birth cohorts in Spain that aims to study the role of environmental pollutants in air, water and diet during pregnancy and early childhood in relation to child growth and development (43). Mothers were enrolled at week 12 of pregnancy from 1997 to 2008 in seven regions of Spain (Flix, Granada, Menorca, Asturias, Gipuzkoa, Sabadell and Valencia). The cohort consisted of 3768 children at birth. During the follow-up visits information on environmental exposures and health outcomes (reproductive, growth and obesity, lung function, allergies and neurodevelopment) were assessed through questionnaires, biomarker measurements, clinical data, and physical exploration. The study website contains details of the design and data available in INMA project (<http://www.proyectoinma.org/>). The study was approved by the Ethical Committees of each participating centre and written consent was obtained from parents.

DNA methylation data assessed with the Infinium HumanMethylation450 BeadChip is available in Sabadell subcohort for 391 and 209 blood samples collected at birth and at age 4 years, respectively (185 paired samples). Furthermore, placental methylation is available for 181 children from Asturias, Gipuzkoa, Sabadell, and Valencia subcohorts. The overlap between placental samples and blood samples is minimal. Cord blood and whole blood collected at age 4y was extracted using the Chemagen kit (Perkin Elmer). DNA concentration was determined by a NanoDrop spectrophotometer (Thermo Scientific) and with the Quant-iT PicoGreen dsDNA Assay Kit (Life Technologies). Placental biopsies were obtained from the inner region of the fetal site of the placenta, and genomic DNA was extracted with the DNeasy® Blood and Tissue Kit (Qiagen) as described elsewhere (44). Blood methylation data was produced in two laboratories: the Genome Analysis Facility of the University Medical Center Groningen (UMCG) in Holland as part of the MeDALL project (0y and 4y), and the Bellvitge Biomedical Research Institute (IDIBELL) in Barcelona as part of the BREATHE project (0y). Placental methylation was measured at the UMCG laboratory. Both laboratories randomized the samples in batches and followed the Illumina protocol for the Infinium HumanMethylation450 BeadChip. Briefly, 500 ng of DNA was bisulfite-converted using the EZ 96-DNA methylation kit, and DNA methylation was measured through hybridization on the BeadChips. BeadChips were scanned with an Illumina iScan and image data was uploaded into the Methylation Module of Illumina’s analysis software GenomeStudio, and converted in β-values. Methylation data from blood and from placenta was quality controlled separately. Two blood samples with overall low quality (MethylAid package) (45), and three blood samples discordant for sex were removed (shinyMethyl package) (10). After applying a stringent detection *P*-value of 1.10E-16 (46), 18 blood samples with a call rate <98% were excluded. Data was normalized with the functional normalization method implemented in the minfi package (24). 7136 probes with a call rate <95%, control probes and probes designed to detect genetic polymorphisms were removed. ComBat was applied to eliminate laboratory batch effects, without removing age differences by keeping age in the statisical model (47). Finally, one of the 12 duplicated samples was excluded. The final dataset consisted of 476 946 probes and 616 samples (391 at age 0y and 209 at age 4y, 185 of them paired 0-4y). The WateRmelon package was used to perform the quality control of the placental DNA methylation data (7). Three samples were excluded due to overall low quality or because they had a call rate <99% (detection *P*-value < 0.05). Ten duplicates were included in the study showing high correlations, and one of them was kept at random. Nine samples were excluded due to sex discrepancies. 1859 probes were eliminated due to their low performance according to the WateRmelon filters. We also removed control probes, probes designed to detect genetic polymorphisms, probes in sexual chromosomes, probes that mapped ambiguously to the genome, and probes containing SNPs as suggested elsewhere (6). Normalization of raw data was done with the dasen method. The final dataset consisted in 181 samples and 433 131 CpG sites in autosomic chromosomes. More details can be found elsewhere (44).

**Inner City Asthma Consortium (ICAC) EPIGEN Cohort**

Our study population consisted of inner-city children aged 6-12 years with atopy and persistent asthma (cases) and without atopy or asthma (healthy controls). The cases and controls were recruited by six sites of the Inner-City Asthma Consortium (Boston; Washington, DC; Denver; New York; Dallas; and Detroit) from census tracts that contain at least 20% of households below the U.S. government poverty level (48). Cases of asthma were required to meet the following criteria: 1) a physician diagnosis of asthma; 2) persistent or uncontrolled disease as defined by the National Asthma Education and Prevention Program (49); 3) physiologic evidence of asthma (FEV_1_ < 85% predicted, or FEV_1_/FVC ratio < 85% and bronchodilator responsiveness (≥ 12%), or PC_20_ < 8 mg/ml of methacholine); and 4) positive prick skin-test to as least one of a panel of indoor aeroallergens (i.e. dust mite, cockroach, mold, cat, dog, rat, or mouse). Controls were required to have: 1) no medical history of asthma, rhinitis, sinusitis, and atopic dermatitis; 2) an FEV_1_ > 85% predicted; and 3) no positive prick skin-tests. The validation population consisted of 101 African Americans between 6 and 12 years of age with atopic asthma (as defined above) that were collected by the Inner-City Asthma Consortium independent of the primary study population.

Peripheral blood mononuclear cells (PBMCs) were isolated from whole blood using the Ficoll density gradient separation. DNA and RNA were isolated simultaneously from the PBMCs using the AllPrep DNA/RNA kit (Qiagen, Germantown, MD), DNA and RNA samples were quantified and purity assessed using a NanoDrop spectrophotometer (Thermo Scientific, Wilmington, DE). RNA integrity was determined using the Bioanalyzer (Agilent, Santa Clara, CA).

To measure methylation at approximately 485 000 single CpG sites across the genome, we used Illumina’s Infinium Human Methylation 450k BeadChip on bisulfite-treated samples. 0.85-1.00 µg DNA were bisulfite converted using the Zymo EZ DNA Methylation kit (Zymo Research, Orange, CA). Each conversion assay included a commercially available positive and negative control sample. Bisulfite converted samples formed the input for the Illumina Infinium Methylation assay using the Human Methylation 450k BeadChips (Illumina Inc, San Diego, CA). The labeling, hybridization, and scanning procedures were performed on the iScan system. All samples were assayed once (no technical replicates) with 194 arrays performed in 3 batches. We first examined the quality control figures created by the minfi R package for the 450k data (50). We observed a strong bi-modal distribution of methylation values in the 450k data as has been previously observed (21). Further data quality for Illumina 450k was assessed using principal components analysis (PCA). The principal components were examined for correlation with all clinical/demographic and laboratory data to identify observable batch effects or covariates that explained variation (51). As a result of PCA, we removed one outlier sample from the 450k methylation data. No laboratory variables were significantly correlated with principal components in the methylation dataset. Out of all demographic and clinical data, age and gender are strongly associated with top PCs in the methylation dataset. Allergic asthma-associated DNA methylation changes were previously reported (52).

***Isle of Wight 1989 Birth Cohort (IoW F1) and Isle of Wight 3^rd^ Generation Cohort (IoW F2)***

A whole population birth cohort was established on the Isle of Wight, UK, in 1989 to prospectively study the natural history of allergic diseases from birth onwards. Both the Isle of Wight and the study population are 99% Caucasian. Ethics approvals were obtained from the Isle of Wight Local Research Ethics Committee (now named the National Research Ethics Service, NRES Committee South Central – Southampton B) at recruitment and for the 1, 2, 4, 10, 18 and 27-year follow-ups. Of the 1536 children born between January 1, 1989, and February 28, 1990, written informed consent was obtained from parents to enroll 1456 newborns. Children have been followed up at the ages of 1 (n = 1167), 2 (n = 1174), 4 (n = 1218), 10 (n = 1373), 18 years (n = 1313) and 27 years (n=1033). Epigenome -wide DNA methylation has been measured in blood derived DNA of 480 subjects at age 18 (~2:1 F:M), and for a subset of these subjects (N=333) additionally in blood DNA at age 10. Analysis in perinatal blood DNA from Guthrie cards is currently in progress.

From January 2012 to February 2017, we have further recruited 1989-1990 cohort participants and 420 newborns of these participants to date termed the Isle of Wight 3^rd^ Generation cohort. Further recruitment to the 3^rd^ generation cohort and follow-up of the offspring is ongoing. Epigenome-wide DNA methylation has measured in 192 Caucasian children, using DNA extracted from cord blood, and for 45 children using DNA extracted from Guthire cards.

For all subjects 500-1000ng DNA per sample underwent bisulfite conversion using the EZ-96 DNA Methylation kit (Shallow) (Zymo Research Corporation, Irvine, USA). Samples were plated onto 96-well plates in random order. The samples were processed with the Illumina Infinium HumanMethylation450 BeadChip (Illumina Inc., San Diego, USA) or Illumina Infinium HumanMethylation EPIC as detailed in Table 1.

Methylation data were pre-processed using the Bioconductor IMA (Illumina methylation analyzer) package accompanied by the ComBat package for quantile normalization, background adjustment, peak correction, as well as as for adjustment of inter-array and batch variations (47, 53). In later samples, the CPACOR (46) pipeline was used for QC and normalisation of the data. Methylation markers on 65 single nucleotide polymorphism (SNP) and sex chromosomes were removed. We applied Illumina background Correction to all intensity values. Any intensity values having detection *P*-values >= 10-16 were set as missing data. Samples with call rate < 98% were excluded.   A quantile normalisation was applied using limma on intensity values  of the remaining sites separately based on six different probe-type categories (Type-I M red, Type-I U red, Type-I M green, Type-I U green, Type-II red, and Type-II green). Beta values were then calculated from these normalised intensity values. The R package ComBat (47) built upon an empirical Bayes framework was used to remove batch effects

***Norwegian Mother and Child Cohort Study (MoBa)***

Participants represent three subsets of mother-offspring pairs from the national Norwegian Mother and Child Cohort Study (MoBa) (54-56). The years of birth for MoBa participants ranged from 1999-2009. MoBa mothers provided written informed consent. Each subset is referred to here as MoBa1, MoBa2, and MoBa3. MoBa1 is a subset of a larger study within MoBa that included a cohort random sample and cases of asthma at age three years (57). We previously reported an association between maternal smoking during pregnancy and differential DNA methylation in MoBa1 newborns (58). We subsequently measured DNA methylation in additional newborns (MoBa2) in the same laboratory (Illumina, San Diego, CA) (59). MoBa2 included cohort random sample plus cases of asthma at age seven years and nonasthmatic controls. MoBa3 was designed to evaluate the association between differential cord blood DNA methylation and later childhood cancer status. Methylation measurements for MoBa3 were made at IARC. Years of birth were 2002-2004 for children in MoBa1, 2000-2005 for MoBa2, and 2000-2008 for MoBa3. All three studies were approved by the Regional Committee for Ethics in Medical Research, Norway. In addition, MoBa1 and MoBa2 were approved by the Institutional Review Board of the National Institute of Environmental Health Sciences, USA.

Details of the DNA methylation measurements and quality control for the MoBa1 participants were previously described (58) and the same protocol was implemented for the MoBa2 participants. Briefly, umbilical cord blood samples were collected and frozen at birth at -80**°**C. All biological material was obtained from the Biobank of the MoBa study (55). Bisulfite conversion was performed using the EZ-96 DNA Methylation kit (Zymo Research Corporation, Irvine, CA) and DNA methylation was measured at 485577 CpGs in cord blood using Illumina Infinium HumanMethylation450 BeadChip (Illumina Inc., San Diego, USA) (3). Raw intensity (.idat) files were handled in R using the *minfi* package19 to calculate the methylation level at each CpG as the beta-value (β=intensity of the methylated allele (M)/(intensity of the unmethylated allele (U) + intensity of the methylated allele (M) + 100)) and the data was exported for quality control and processing. Probe and sample-specific quality control was performed in the MoBa1, MoBa2, and MoBa3 datasets separately. Similar protocols were applied to MoBa1 and Moba2, as follows: Control probes (N=65) and probes on X (N=11 230) and Y (N=416) chromosomes were excluded in both datasets. Remaining CpGs missing > 10% of methylation data were also removed (N=20 in MoBa1, none in MoBa2). Samples indicated by Illumina to have failed or have an average detection p value across all probes < 0.05 (N=49 MoBa1, N=35 MoBa2) and samples with gender mismatch (N=13 MoBa1, N=8 MoBa2) were also removed. For MoBa1 and MoBa2, we accounted for the two different probe designs by applying the intra-array normalization strategy Beta Mixture Quantile dilation (BMIQ) (11). The Empirical Bayes method via *ComBat* was applied separately in each dataset for batch correction using the *sva* package in *R* (47)*.* After quality control exclusions, the sample sizes were 1068 for MoBa1 and 685 for MoBa2. For MoBa3, samples were completely randomized, and bisulfite conversion and methylation measurements were performed by the Epigenetics Group at IARC (Lyon, France). Similar data quality control and processing was applied with some slight differences. Methylation features were filtered from (i) cross-reactive probes, (ii) probes mapping to sex chromosomes and (iii) probes overlapping with a known single nucleotide polymorphism (SNP) with an allele frequency of at least 5% in the overall population (all ethnic groups), resulting in the exclusion of 36 231 probes. Data quality was further assessed using box plots for the distribution of methylated and unmethylated signals, and multidimensional scaling plots and unsupervised clustering were used to check for sample outliers. After background correction and color-bias adjustment, type I and type II probe distributions were aligned using the intra-sample BMIQ normalization (11) from the watermelon package. Batch effects were corrected by *sva* (60). After quality control, the sample size for MoBa3 was 253.

***Norway Facial Clefts Study (NCL)***

The Norway Facial Clefts Study (NCL) is a national population-based case-control study of cleft lip and cleft palate, disorders characterized by the incomplete fusion of the lip and/or palate during development. The study design has been previously described in detail (61). Study approval was obtained by the Norwegian Data Inspectorate and Regional Medical Ethics Committee of Western Norway and informed consent was provided by both the mother and father. Briefly, between the years of 1996 and 2001 all families of newborns referred for cleft surgery in Norway were contacted and, of those eligible, 88% agreed to participate (N=573). Controls were selected by a random sampling of roughly 4 per 1000 live births in Norway during that same time period and, of those eligible, 76% agreed to participate (N=763).

Epigenome-wide DNA methylation was measured in 889 newborns, using DNA extracted from heel stick blood samples that were collected 2-3 days after delivery as part of a standardized program of testing for phenylketonuria (PKU). A detailed description of DNA methylation data generation (Illumina Infinium HumanMethylation450 BeadChip (Illumina Inc., San Diego, USA)), quality control, and data pre-processing has been provided previously (3, 62). Briefly, one microgram of DNA was bisulfite converted using the EZ DNA Methylation kit following the manufacturer’s protocol. 898 newborn and 60 technical control samples were run on Illumina Infinium HumanMethylation450 BeadChip according to the manufacturer’s instructions at the NIH Center for Inherited Disease Research. After exclusions, 889 samples remained for analysis. Raw intensity data were obtained using the Illumina GenomeStudio methylation module (version 2011.1). At each CpG site on the array, methylation status was determined based on intensity measures corresponding to unmethylated (U) or methylated (M) signal. The Illumina HumanMethylation450 BeadChip contains two probe types: Infinium Type I (2 probe types, 1 color channel) and II (1 probe type, 2 color channels). As the Type II probes use two colour channels to assess methylation, dye bias was corrected using the normalization function (normalizeMethyLumiSet), provided in the R package, methylumi (63). Before association analysis, the M and U intensity values for Type I and II probes were separately background adjusted using the ENmix method (64) and quantile normalized using the normalization function (normalize.quantiles), provided in the R package, Affy (65). The probe design type bias between type I and II probes was corrected using the RCP method (66). The β-value (M/(M+U+100)) was then computed and used in the association analysis. β-values that were more than 3 standard deviations from the mean and methylation levels that were deemed undetectable (Illumina detection *P*-value ≥ 0.05) were excluded.

***Newborn Epigenetics Study (NEST)***

NEST is a multiethnic birth cohort designed to identify the effects of early exposures on epigenetic profiles and phenotypic outcomes (67). Pregnant women were recruited from prenatal clinics serving Duke University Hospital and Durham Regional Hospital Obstetrics facilities in Durham, North Carolina from April 2005 to July 2009. Gestational age at enrollment ranged from 6 to 42 weeks (median 30 weeks). Eligibility criteria were women aged 18 years or older, English speaking, pregnant, and an intention to use one of the two obstetrics facilities. Among these, women infected with HIV or intending to give up custody of the offspring of index pregnancy were excluded. Current smokers were targeted for the first ~200 participants. Of the 1101 women who met eligibility criteria and were approached, 895 (81%) were enrolled and umbilical cord blood was collected from 741 infants. The current analysis was limited to the 413 infants with 450k and covariate data. This study was approved by the Duke Institutional Review Board.

Genomic DNA from buffy coat specimens was extracted from umbilical cord blood using Puregene Reagents (Qiagen, Valencia, CA). Bisulfite conversion was performed using the EZ-96 DNA Methylation Kit (Zymo Research Corporation) and DNA methylation was measured at 485 577 CpGs using Illumina Infinium HumanMethylation450 BeadChip (Illumina Inc., San Diego, USA) (3). Illumina’s GenomeStudio Methylation module version 1.0 (Illumina Inc.) was used to calculate the methylation level at each CpG as the beta value. Probe and sample-specific quality control was performed in the NEST cohort using a similar approach to MoBa1 and MoBa2 cohorts as the data analysis was completed at the NIEHS. Specifically, control probes (N=65) and probes on X (N=11 230) and Y (N=416) chromosomes were excluded as well as CpGs missing > 10% of methylation data. Samples indicated by Illumina to have failed or have an average detection *P*-value across all probes < 0.05 and samples with gender mismatch were also removed. The two different probe designs by applying the intra-array normalization strategy Beta Mixture Quantile dilation (BMIQ) (11). The Empirical Bayes method via *ComBat* was applied for batch correction using the *sva* package in *R* (47)

***Northern Finland Birth Cohorts (NFBC), 1966 and 1986***

The cohorts of mothers and newborns were collected at 20-year intervals from the provinces of Oulu and Lapland: the older cohort with an expected date of birth in 1966, comprising of 12 068 deliveries and 12 231 children (NFBC 1966) and the younger cohort with an expected date of birth between 1.7.1985- 30.6.1986, comprising 9362 mothers and 9479 children (NFBC 1986). For both cohorts interviews and postal questionnaires were completed/returned from the 24th gestational week onwards (data since 12-16th gestational week). The course of pregnancy and delivery, including complications, were confirmed from patient records, as was the neonatal outcome. The children were followed-up at the ages of 6-12 months, 7-8 years (NFBC 1986), 14-16 years (NFBC 1966, 1986) and at the age of 31 and 46 (NFBC 1966). Follow-up of the NFBC 1986 with clinical data collection -- similarly as for the NFBC 1966 at age 31-- was carried out during 2001-2003. The data have been supplemented by various hospital records and statistical register data. The Data Protection Ombudsman of Finland has reviewed the NFBC program, the ethical committee of Northern Ostrobotnia Hospital District has approved the program and the permission from the Finnish Ministry of Social Affairs and Health was obtained for the use of register data and patient records.

As part of the EuroHEALTHaging, EGEA and DynaHEALTH project, the Illumina Infinium HumanMethylation450 BeadChip and EPIC BeadChip (Illumina Inc., San Diego, USA) have been used to generate epigenetic data on adult samples at age 16 (NFBC1986; N=580), 31y (NFBC1966; N=780) and 46y (NFBC1966; N=780) (3). The sample has been gathered from a random sample where full set of information at follow-up is available with aim to explore the contribution of DNA methylation in the incidence on long-term adult diseases from a life course perspective.

***New Hampshire Birth Cohort Study (NHBCS)***

NHBCS is an ongoing prospective longitudinal birth cohort that began in 2009 and includes over 1500 mother-infant pairs from the Concord and Lebanon regions in New Hampshire, USA. All enrolled mothers provided written informed consent and the study has been approved by the Institutional Review Board. Pregnant mothers were recruited if they were literate in English, mentally competent, between 18–45 years old, received prenatal care at one of the study-clinics, and reported using a private, unregulated well as the primary source of home drinking water without plans to move prior to delivery. Infants included in the cohort were singleton pregnancies. Pre- and post-delivery questionnaires and medical record reviews were administered to collect sociodemographic, lifestyle, and clinical data, and multiple environmental and biological samples were obtained.

After delivery, cord blood (n=135) and placental tissue (n=343) were sampled for DNA methylation analyses. DNA was extracted then bisulfite converted using the EZ DNA Methylation kit, semi-randomly distributed among plates to limit batch effect bias, then subjected to the Illumina Infinium HumanMethylation450 BeadChip (Illumina Inc., San Diego, USA) for epigenome-wide DNA methylation measurement (3). Quality control and pre-processing were conducted in the minfi package in R. The control probes were utilized to evaluate the quality of our samples, poor bisulfite conversion or color-specific issues for each array. Probes with detection *P*-values > 0.01 in at least one sample were removed. Data were processed via functional normalization (10) followed by beta-mixture quantile normalization (BMIQ) to correct for probe-type bias (11) and batch effect removal via combat (47).

***Prevention and Incidence of Asthma and Mite Allergy (PIAMA)***

The PIAMA study is a birth cohort study of children born between 1996-1997. Details of the study design have been published previously (68). 10 232 pregnant women completed a validated screening questionnaire at their prenatal health care clinic (n=52). Based on this screening, 7862 women were invited to participate, of whom 4146 women agreed and gave informed consent. Mothers reporting a history of asthma, current hay fever or allergy to pets or house dust mite were defined as allergic. Children were recruited during the first trimester of pregnancy. Follow-up of the children took place at 3 months of age and yearly from 1 to 8 years of age DNA was extracted of a children who provided a blood sample at age 4 and 8 years. The Medical Ethical Committees of the participating institutes approved the study, and all participants gave written informed consent. The response rates to the annual questionnaires ranged from 3764 at age 1 to 3269 at age 8 years.

PIAMA has samples of 4 year, 8 year and 16 years blood DNA methylation data, and 4 year and 8 years sample are from MeDALL (Mechanisms of the Development of ALLergy) study (69). MeDALL is a collaborative project supported by the European Union under the Health Cooperation Work Programme of the 7th Framework programme (grant agreement number 261357). At age 16, also DNA from nasal brushing was isolated. In the MEDALL study, peripheral blood samples were collected from all consenting cohort participants, and DNA from peripheral and cord blood samples was isolated by the laboratories participating in the MEDALL study using different methods. To uniform the concentration and purity the samples underwent a precipitation-based concentration and purification using GlycoBlue (Ambion) if needed. DNA concentration was determined by Nanodrop measurement and picogreen quantification. After normalization of the concentration, the samples were randomized to avoid batch effects. Standard male and female DNA samples were included in this step for control reasons. 500 ng of DNA of each sample was bisulfite-converted using the EZ 96-DNA methylation kit following the manufacturer’s standard protocol. After verification of the bisulfite conversion using Sanger Sequencing, the DNA methylation was measured using the Illumina Infinium HumanMethylation450 BeadChip (Illumina Inc., San Diego, USA) (3). A series of steps were completed for quality control and data analysis. First, we implemented sample filtering to remove bad quality and mixed up samples. Second, we filtered out the probes to remove the CpG sites which are not mapped to unique location on the genome and CpGs containing single nucleotide polymorphisms (SNPs) at the target site (6). Third, we implemented “DASEN” to perform signal correction and normalization (7). Fourth, to remove bias in methylation profiles unrelated to underlying biological processes, we implemented a correction procedures based on 613 negative control probes presented in 450K arrays since these negative control probes are supposed to not relate to biological variation (70). Finally, we implemented PCA on control probes data, then, we performed 10 000 permutation for controls probes data and selected principal components with *P*-value defined as to get the p value of (number of var(random pc) > var(pc))/ (number of permutations) <10^-4^. The methylation data for each CpG are thus the residuals from a linear model fitting incorporating the significant 5 PCs.

***Piccoli+***

Piccoli+ is a multicentric Italian birth cohort that recruited 3338 new-borns and their mothers in 5 centres: Turin, Trieste, Florence, Viareggio and Rome between 2011 and 2015, ([www.piccolipiu.it](http://www.piccolipiu.it)) (71). Mothers were contacted 6, 12, 24 and 48 months after delivery to collect follow-up infomation using questionnaires, and children underwent a medical examination at 4 years of age. Growth trajectories, neurocognitive test-results and data on respiratory health are available. Ethical approvals were obtained from the Ethics committees of the Local Health Unit Roma E (management centre), of the Istituto Superiore di Sanità (National Institute of Public Health) and of each local centre.

A set of 99 children from the Turin centre is included in the EXPOsOMICS Children Studies, for which data on cord blood DNA methylation is available. Aliquots of cord blood samples (collected and frozen at birth at -80**°**C) were shipped on dry ice to the Epigenetics Group at IARC, Lyon, France, where DNA was extracted (QIAamp 96 DNA Blood Kit, Qiagen 51161), quantified (Quant-iT PicoGreen dsDNA Assay Kit, Molecular Probes P7589) and bisulfite converted (600 ng of DNA using EZ-96 DNA Methylation kit, Zymo Research D5004). Samples were completely randomized, and DNA methylation was measured at 485 577 CpGs using Illumina Infinium HumanMethylation450 BeadChip (Illumina Inc., San Diego, USA) (3). Raw intensity (.idat) files were handled in R using the *minfi* package to calculate the methylation level at each CpG as the beta-value (β=intensity of the methylated allele (M)/(intensity of the unmethylated allele (U) + intensity of the methylated allele (M) + 100)), and the data was exported for quality control and processing. Methylation features were filtered from cross-reactive probes and low-quality probes (probes having bead counts < 3 in at least 5% of samples). Data quality was further assessed using box plots for the distribution of methylated and unmethylated signals, and multidimensional scaling plots and unsupervised clustering were used to check for sample outliers and potential gender mismatches. Samples having >1% of CpG sites with a detection *P*-value > 0.05 were removed. After background correction and color-bias adjustment, type I and type II probe distributions were aligned using the intra-sample BMIQ normalization (11). To correct for technical confounding, chip number (Sentrix_ID) and sample position on the chip (Sentrix_Position) were included in every regression model. After quality control, the sample size of processed Piccoli+ samples remained 99.

***The Prediction and Prevention of Preeclampsia and Intrauterine Growth Restriction Study (PREDO)***

PREDO is a prospective birth cohort study of Finnish women who were pregnant between 2005 and 2009 and their children. The PREDO study cohort was set up to identify novel risk factors and biomarkers in pregnant women associated with the development of preeclampsia and intrauterine growth restriction (IUGR), to (a) identify effective methods for prediction and prevention of preeclampsia in at-risk women, and (b) determine the association between exposure to preeclampsia, IUGR, or their risk factors and child developmental/health outcomes. Women with a singleton, intrauterine pregnancy who visited antenatal clinics at ten study hospitals in Finland for their first ultrasound screening at 12+0-13+6 weeks+days of gestation were recruited in the PREDO study. Two groups of pregnant women were enrolled: first, pregnant women with a known clinical risk factor status for preeclampsia and IUGR, and second, pregnant women who volunteered to participate regardless of their risk factor status for preeclampsia and IUGR. The sample with a known risk factor status comprises 1079 pregnant women who gave live birth (969 of these women had at least one and 110 had none of the known risk factors for preeclampsia and IUGR). The community-based sample comprises 3698 pregnant women who gave live birth. The sample with a known risk factor status visited antenatal clinics up to four times during pregnancy and both samples filled in bi-weekly self-reports. The post-delivery follow-up has taken place at approximately 2 weeks, 6 months, and 3.5 years after the delivery. The most recent follow-up started in 2016 and is ongoing. The study protocol was approved by the Ethics Committee of Obstetrics and Gynaecology, and Women, Children and Psychiatry of the Helsinki and Uusimaa Hospital District and by the participating hospitals. All participants provided written informed consent. Consent of participating children were provided by parent(s)/guardian(s). Details of the study design, inclusion criteria, enrollment and data collection are described elsewhere (72).

In the high-risk sample, cord blood samples were collected according to standard procedures. DNA was extracted at the National Institute for Health and Welfare, Helsinki, Finland and and the Finnish Institute of Molecular Medicine, University of Helsinki, Finland and methylation analyses were performed at the Max Planck Institute in Munich, Germany. DNA was bisulphite-converted using the EZ-96 DNA Methylation kit (Zymo Research). Genome-wide methylation status of over 485 000 CpG sites was measured using the Illumina Infinium HumanMethylation450 BeadChip (Illumina Inc., San Diego, USA) according to the standard protocol in 876 samples (analyses of 140 samples with Illumina EPIC array are ongoing in June 2016) (3). The arrays were scanned using the iScan System (Illumina Inc., San Diego, USA). The quality control pipeline was set up using the R-package minfi. Samples were excluded if they were duplicates, outliers in the median intensities, and because of sex discrepancy. Furthermore, any probes on chromosome X or Y, cross-hybridizing probes as well as probes containing SNPs, and CpGs with a detection *P*-value > 0.01 in at least 50% of the samples were excluded. The final dataset contains 428 619 CpGs and 834 samples. Methylation beta-values were normalized using the funnorm function and incorporating the first ten principal components from the internal control probes. To check for batch effects, principal components were computed on these beta values. Two batches, i.e. slide and well, were significantly associated to the first principal component. These batch effects were removed iteratively using the Combat package.

***PRISM***

The PRogramming of Intergenerational Stress Mechanisms (PRISM) study, is a prospective pregnancy cohort of mother-child pairs originally designed to examine how perinatal stress influences respiratory health in children. Procedures were approved by the Institutional Review Boards at the Brigham and Women’s Hospital (BWH) and the Icahn School of Medicine at Mount Sinai. Beth Israel Deaconess Medical Center (BIDMC) relied on BWH for review and oversight of the protocol. Written consent was obtained from all participants. Women were recruited from prenatal clinics during the first or second trimester (<28 weeks gestation) from the Beth Israel Deaconess Medical Center (BIDMC) and the East Boston Neighborhood Health Center in Boston Massachusetts, USA, from March 2011 to August 2012 and from the Icahn School of Medicine at Mount Sinai in New York, New York, USA, starting in April 2013 where collections are still underway. Recruitment sites were chosen to ensure desired heterogeneity in sociodemographic and racial/ethnic characteristics. Eligibility criteria included: (i) English- or Spanish-speaking; (ii) age ≥18 years at enrollment; and (iii) singleton pregnancy.

Cord blood, placental samples (fetal side), and umbilical artery DNA was isolated using Qiagen Tissue DNA extraction kits (Qiagen, Valencia CA) and quantified using an Implen Nanophotometer Pearl (Westlake Village, CA). 500ng of DNA was bisulfite-treated using the EZ DNA Methylation-Gold™ Kit (Zymo Research, Orange, CA) and analyzed by the Illumina Infinium HumanMethylation450 BeadChip (Illumina Inc., San Diego, USA) (3). Samples were arranged on chips and plates with a stratified randomization followed by statistical checks for balance on birthweight z-score, gestational age, sex, and city of collection. The presence of failed arrays or outliers was checked with detection *P*-values (all samples passed with detection *P*-values <0.05 in >99% of probes) and through visualization of principal components analysis (PCA). Potential batch effects were further assessed with five pairs of technical replicates per tissue that were arranged across chips and plates. Sample identity was checked via imputed sex and agreement of genotype with paired tissues. Probes with detection *P*-values >0.05 or beadcount <3 in >1% of samples were dropped using the wateRmelon pfilter function. Data were preprocessed using background correction(23), dye bias and probe type adjustment(9). BMIQ (Beta Mixture Quantile dilation) intra-sample normalization was applied to all probes to adjust the methylation values of Infinium II probes into a statistical distribution characteristic of Infinium I probes.

***Project Viva***

Project Viva is a prospective pre-birth cohort of mothers and their children recruited from a multispecialty group practice in Eastern Massachusetts, USA, which has been described in detail elsewhere (73). The Institutional Review Board of Harvard Pilgrim Health Care approved the study and participating women provided written informed consent. Women were enrolled from 1999 to 2002 and enrollment included a total of 2128 live births. Follow up of the children through adolescence is ongoing. Trained medical personnel obtained venous umbilical cord blood samples immediately after delivery, which they promptly stored in a dedicated refrigerator (4ºC) and transported for processing within 24 hours. A similar protocol was implemented for peripheral venipuncture blood samples obtained at early childhood (median 3.2 years) and mid-childhood (median 7.7 years). Trained laboratory staff processed the samples on the same day, and extracted DNA by using the Qiagen Puregene Kit (Valencia, CA). Aliquots were then stored at -80ºC until analysis.

We arranged DNA samples using a stratified randomization to ensure balance of cohort characteristics across sample plates/batches. Samples were bisulfite converted using the EZ-96 DNA Methylation kit (Zymo Research Corporation, Irvine, USA). Illumina FastTrack Microarray Services (San Diego, CA) performed the analyses using the Illumina Infinium HumanMethylation450 BeadChip (Illumina Inc., San Diego, USA) (3). Failing samples were rerun and passing arrays were defined as having >99% of probes with a detection *P*-value <0.05. After excluding samples with identity concerns (inconsistent genotyping and/or inferred sex) 485 unique samples are available for cord blood analyses, 120 samples for early-childhood analyses, and 460 for mid-childhood analyses. Standard sample preprocessing includes the exclusion of allosomal probes, non-CpG probes, and failing probes (<99% of samples with detection *P*-values <0.05). Further pre-processing and normalization steps typically include 1.Background subtraction using the out-of-band probes (noob); 2. Dye bias adjustment; 3. Within-array type II probe adjustment using BMIQ; and 4. Adjustment for batch effects (e.g. plate) using methods such as ComBat.

***The Western Australian Pregnancy Cohort (Raine) Study***

The Western Australian Pregnancy Cohort (Raine) Study (http://www.rainestudy.org.au) is a longitudinal Australian birth cohort that has serially assessed the offspring of 2900 pregnant women from 18 weeks gestation in utero. Follow-up of the offspring has been undertaken at 1, 2, 3, 5, 8, 10, 14 and 17, 24 years (74, 75).

DNA was extracted from whole blood samples obtained at 17 year old follow up. Bisulphite conversion was prepared from whole blood cells by standard phenol:chloroform extraction and ethanol precipitation. Processing of the Illumina Infinium HumanMethylation450 BeadChips (Illumina Inc., San Diego, USA) was carried out by the Centre for Molecular Medicine and Therapeutics (CMMT) <http://www.cmmt.ubc.ca> (3). The raw IDAT files were imported into R using the rnb.run.import() function available in the *RnBeads* package. Two packages were used to perform quality control checks of the samples; *shinyMethyl* and *MethylAid*. Three samples were evident as outliers based on the output from *shinyMethyl* and *MethylAid*. Gender was inferred using the rnb.execute.gender.prediction() function available in the *RnBeads* package. When predicted gender was compared to known gender there was a single discrepancy. Fifty-eight of the samples were run in duplicate or triplicate and the 65 SNP probes present on the BeadChip were used to assess genetic similarity between these individuals as a check for sample mix-ups. The rnb.plot.snp.heatmap() function available in the *RnBeads* package was used to produce a heatmap of *β* values. One contaminated sample was excluded based on this plot. Intentional SNP probes (n=65), sex chromosome probes (n=11 648) and probes with a detection *p*-value greater than 0.05 in any sample (n=10 777) were removed. A further 160 probes with low bead counts (bead counts less than 3 in more than 5% of samples) were removed.

***Rhea Mother-Child Cohort (Rhea)***

The mother-child “Rhea” study in Crete (www.rhea.gr) is a prospective cohort examining a population sample of pregnant women and their children, at the prefecture of Heraklion (n=1500). Pregnant women were recruited at the time of the first comprehensive ultrasound examination, around week 12 of gestation, from four prenatal clinics (two public and two private) in Heraklion city that cover the wider Heraklon region, during the twelve-month period from February 2007 until February 2008. Their children have been followed up until age 7-8. The study aims are to evaluate (a) nutritional, environmental, biological and psychosocial exposures in the prenatal period and in early childhood, (b) the association of these exposures with the development of the foetus and the child, (c) mother’s health during and after pregnancy, and (d) genetic susceptibility and the interactions between genetic and environmental factors affecting child health. At each visit written informed consent was obtained from the mothers, and the Ethics Committee of the University Hospital at Heraklion approved the study protocols.

A set of 100 children from the Rhea-cohort is included in the EXPOsOMICS Children Studies, for which data on cord blood DNA-methylation is available. Aliquots of cord blood samples (collected and frozen at birth at -80**°**C) were shipped on dry ice to the Center for Research in Environmental Epidemiology (CREAL), Barcelona, Spain, where DNA was extracted and quantified (Quant-iT PicoGreen dsDNA Assay Kit, Molecular Probes P7589). DNA samples were then completely randomized and bisulfite converted (600 ng of DNA using EZ-96 DNA Methylation kit, Zymo Research D5004) at the Epigenetics Group at IARC, Lyon, France, where DNA methylation was measured at 485 577 CpGs using Illumina Infinium HumanMethylation450 BeadChip (Illumina Inc., San Diego, USA) (3). Raw intensity (.idat) files were handled in R using the *minfi* package to calculate the methylation level at each CpG as the beta-value (β=intensity of the methylated allele (M)/(intensity of the unmethylated allele (U) + intensity of the methylated allele (M) + 100)), and the data was exported for quality control and processing. Methylation features were filtered from cross-reactive probes and low-quality probes (probes having bead counts < 3 in at least 5% of samples). Data quality was further assessed using box plots for the distribution of methylated and unmethylated signals, and multidimensional scaling plots and unsupervised clustering were used to check for sample outliers and potential gender mismatches. Samples having >1% of CpG sites with a detection *P*-value > 0.05 were removed. After background correction and color-bias adjustment, type I and type II probe distributions were aligned using the intra-sample BMIQ normalization (11). To correct for technical confounding, chip number (Sentrix_ID) and sample position on the chip (Sentrix_Position) were included in every regression model. After quality control, the sample size of processed Rhea samples remained 100.

***Rhode Island Child Health Study (RICHS)***

RICHS enrolled mother-infant pairs (n=840) from 2009 through 2014 following delivery at the Women and Infants Hospital (Providence, RI, USA). All enrolled mothers provided written informed consent and the study has been approved by the Institutional Review Board. Infants born large or small for gestational age were oversampled, then matched (on gestational age, infant sex, and maternal age) to infants appropriate for gestational age. Only term (>37 week gestation), singleton, viable infants without indication of congenital or chromosomal abnormalities, born to mothers with good physical and psychological health between the ages of 18-40 years were included. After delivery but prior to discharge, mothers participated in an interviewer-administered structured questionnaire to obtain information on exposures, demographics, and lifestyle factors.

Cord blood (n=450) and placental tissue (n=840) were sampled after delivery; DNA methylation analyses using the Illumina Infinium HumanMethylation450 BeadChip (Illumina Inc., San Diego, USA) were performed on subsets of cord blood (n=96) and placental tissue (n=336) (3). DNA was extracted then bisulfite converted using the EZ DNA Methylation kit and subsequently subjected to the Illumina Infinium HumanMethylation450 BeadChip for epigenome-wide DNA methylation measurement. Samples were semi-randomly distributed among plates to limit batch effect bias. Post-array processing was conducted in the minfi package in R. Array control probes were used to assess the quality of our samples and evaluate potential poor bisulfite conversion or color-specific issues for each array. Probes with detection *P*-values > 0.01 in at least one sample were removed. Data were processed via functional normalization (10) followed by beta-mixture quantile normalization (BMIQ) to correct for probe-type bias (11). Remaining batch effects were corrected for by converting to M-values, adjusting for plate via combat (47), then re-converting the data back to beta values. Removal of batch effect was confirmed with principal components analysis.

***Study to Explore Early Development, Phase I (SEED I)***

The Study to Explore Early Development (SEED) is a multi-site US-based case-control study of autism that has been described in detail (76, 77). SEED phase I, enrolled families with a child born in a study catchment area between September 1, 2003 and August 31, 2006. Enrolled children were aged 30-68 months at the completion of in-person clinical assessments and biospecimen collection. IRB approval was obtained from each of six SEED study sites including Northern California Kaiser Permanente (CA), Johns Hopkins University (MD), University of North Carolina (NC), University of Pennsylvania (PA), University of Colorado Denver (CO), and the Centers for Disease Control (GA) as well as from the Data Coordinating Center site at Michigan State University.

Whole blood genomic DNA was extracted from 980 SEED samples and 500ng was bisulfite treated using the EZ DNA methylation kit (Zymo Research, Irvine, CA). Samples were randomized across and within plates and run on the Illumina Infinium HumanMethylation450 BeadChip (Illumina Inc., San Diego, USA) at the Center for Epigenetics, Johns Hopkins University (3). Each plate contained replicate samples, as well as two internal control samples used by the Epigenetics Center for cross-plate comparisons and quality control measures. Analyses were performed using Bioconductor and R-3.0.x. Illumina idat files were obtained and processed using the minfi package (version 1.8.9) (24). We assessed the correlation of replicate samples across plates to identify problems with particular plates/batches and to assess the accuracy of the DNA methylation values; correlation coefficients for the 22 replicate samples ranged from 0.989 to 0.997. Based on insufficient probe intensity in >10% of samples, 566 probes were excluded. In addition, 28 289 probes with ambiguous mapping have been removed, leaving a total of 456 662 loci for downstream analyses. Six samples with low detection *P*-values in >10% of probes and 3 samples with observed to reported sex-discrepancies were removed, resulting in high quality DNAm data for 971 samples. We performed noob background correction (25) and adjusted data for batch effects using combat in the sva package (version 3.9.1) (27).

***The Swedish Twin study On Prediction and Prevention of Asthma (STOPPA)***

STOPPA is a twin cohort study including n=752 individuals (78). Study participants were selected from an ongoing data collection within the Child and Adolescent Twin study in Sweden (CATSS) based on the pair’s asthma status (79). Approximately one third each of asthma concordant (ACC), asthma discordant (ADC) and healthy concordant (HCC) pairs took part in clinical examinations including questionnaires, lung function testing (spirometry with reversibility test and fractional exhaled nitric oxide, FeNO) and collection of biosamples. The twins were 9-14 years old at the time of invitation to the study. At the clinical examination, n=292 (39%) were in late childhood (9 up to 12 years of age) and n=460 twins (61%) were adolescents (12 years of age and above; the oldest twins were 15 years at the time of examination).

The study population has been linked to the Swedish population-based Medical Birth Register for information on pregnancy and delivery outcomes, the National Patient Register for all in- and outpatient diagnoses and the Swedish Prescribed Drug Register for data on prescribed drugs since 2005. Biosamples include whole blood (collected in 4 ml EDTA tubes and stored at -80°C) from n=708 twins. Further details regarding STOPPA have been provided in a separate publication (78).

DNA was extracted from whole blood using the Chemagic Star 400 kit (PerkinElmer chemagen, Baesweiler, Aachen, Germany) according to a standardized protocol. Samples allocation was performed by complete randomization of samples between analysis plates and chips, with the exception that samples from twin pairs were kept within the same chip to allow for within-pair comparisons free of batch effects. Laboratory analyses took place at the Mutation Analysis Facility (MAF), Karolinska Institutet, Stockholm, Sweden, using the Infinium HumanMethylation450 Beadchip Kit (Illumina, Inc., San Diego, California, USA).

Quality control, sample and probe filtering were performed using *RnBeads* (80). Predicted gender and phenotype-based sex were compared, and matched for all samples. Probes were filtered out due to overlap with single nucleotide polymorphisms or specific nucleotide contexts, unreliable measurements (defined as detection *P*-values > 5^*^10^-8^), or location on sex chromosomes, leaving approximately 455 000 CpG probes for final analyses when using the full data set. The methylation data were normalized using the dasen method, which includes background adjustment and separate between-array normalization of Type I and Type II probes (7). Methylation at each CpG site was expressed as beta values.

To allow for all twins to be retained within the sample, generalized estimating equation (GEE) models are generally used in analyses using STOPPA data. By specifying twin pairs as clusters, the GEE method produces robust standard errors and corrects for within-cluster (i.e. within-pair) correlations. The parameter estimates themselves are not affected. For these analyses the R package *drgee* is used (81).

**REFERENCES**

1. Boyd A, Golding J, Macleod J, Lawlor DA, Fraser A, Henderson J, et al. Cohort Profile: the 'children of the 90s'--the index offspring of the Avon Longitudinal Study of Parents and Children. Int J Epidemiol. 2013;42(1):111-27.

2. Fraser A, Macdonald-Wallis C, Tilling K, Boyd A, Golding J, Davey Smith G, et al. Cohort Profile: the Avon Longitudinal Study of Parents and Children: ALSPAC mothers cohort. Int J Epidemiol. 2013;42(1):97-110.

3. Bibikova M, Barnes B, Tsan C, Ho V, Klotzle B, Le JM, et al. High density DNA methylation array with single CpG site resolution. Genomics. 2011;98(4):288-95.

4. Relton CL, Gaunt T, McArdle W, Ho K, Duggirala A, Shihab H, et al. Data Resource Profile: Accessible Resource for Integrated Epigenomic Studies (ARIES). Int J Epidemiol. 2015;44(4):1181-90.

5. Wickman M, Kull I, Pershagen G, Nordvall SL. The BAMSE project: presentation of a prospective longitudinal birth cohort study. Pediatr Allergy Immunol. 2002;13 Suppl 15:11-3.

6. Chen YA, Lemire M, Choufani S, Butcher DT, Grafodatskaya D, Zanke BW, et al. Discovery of cross-reactive probes and polymorphic CpGs in the Illumina Infinium HumanMethylation450 microarray. Epigenetics. 2013;8(2):203-9.

7. Pidsley R, CC YW, Volta M, Lunnon K, Mill J, Schalkwyk LC. A data-driven approach to preprocessing Illumina 450K methylation array data. BMC Genomics. 2013;14:293.

8. Bousquet J, Anto J, Auffray C, Akdis M, Cambon-Thomsen A, Keil T, et al. MeDALL (Mechanisms of the Development of ALLergy): an integrated approach from phenotypes to systems medicine. Allergy. 2011;66(5):596-604.

9. Ma X, Buffler PA, Wiemels JL, Selvin S, Metayer C, Loh M, et al. Ethnic difference in daycare attendance, early infections, and risk of childhood acute lymphoblastic leukemia. Cancer Epidemiol Biomarkers Prev. 2005;14(8):1928-34.

10. Fortin JP, Labbe A, Lemire M, Zanke BW, Hudson TJ, Fertig EJ, et al. Functional normalization of 450k methylation array data improves replication in large cancer studies. Genome Biol. 2014;15(12):503.

11. Teschendorff AE, Marabita F, Lechner M, Bartlett T, Tegner J, Gomez-Cabrero D, et al. A beta-mixture quantile normalization method for correcting probe design bias in Illumina Infinium 450 k DNA methylation data. Bioinformatics. 2013;29(2):189-96.

12. Eskenazi B, Bradman A, Gladstone EA, Jaramillo S, Birch K, Holland N. CHAMACOS, A Longitudinal Birth Cohort Study: Lessons from the Fields. J Children's Health. 2003;1(1):3-27.

13. Eskenazi B, Harley K, Bradman A, Weltzien E, Jewell NP, Barr DB, et al. Association of in utero organophosphate pesticide exposure and fetal growth and length of gestation in an agricultural population. Environ Health Perspect. 2004;112(10):1116-24.

14. Yousefi P, Huen K, Aguilar Schall R, Decker A, Elboudwarej E, Quach H, et al. Considerations for normalization of DNA methylation data by Illumina 450K BeadChip assay in population studies. Epigenetics. 2013;8(11):1141-52.

15. Yousefi P, Huen K, Dave V, Barcellos L, Eskenazi B, Holland N. Sex differences in DNA methylation assessed by 450 K BeadChip in newborns. BMC Genomics. 2015;16:911.

16. Yousefi P, Huen K, Quach H, Motwani G, Hubbard A, Eskenazi B, et al. Estimation of blood cellular heterogeneity in newborns and children for epigenome-wide association studies. Environ Mol Mutagen. 2015;56(9):751-8.

17. Kirchberg FF, Harder U, Weber M, Grote V, Demmelmair H, Peissner W, et al. Dietary protein intake affects amino acid and acylcarnitine metabolism in infants aged 6 months. J Clin Endocrinol Metab. 2015;100(1):149-58.

18. Koletzko B, von Kries R, Closa R, Escribano J, Scaglioni S, Giovannini M, et al. Lower protein in infant formula is associated with lower weight up to age 2 y: a randomized clinical trial. Am J Clin Nutr. 2009;89(6):1836-45.

19. Rzehak P, Saffery R, Reischl E, Covic M, Wahl S, Grote V, et al. Maternal Smoking during Pregnancy and DNA-Methylation in Children at Age 5.5 Years: Epigenome-Wide-Analysis in the European Childhood Obesity Project (CHOP)-Study. PLoS One. 2016;11(5):e0155554.

20. Weber M, Grote V, Closa-Monasterolo R, Escribano J, Langhendries JP, Dain E, et al. Lower protein content in infant formula reduces BMI and obesity risk at school age: follow-up of a randomized trial. Am J Clin Nutr. 2014;99(5):1041-51.

21. Touleimat N, Tost J. Complete pipeline for Infinium((R)) Human Methylation 450K BeadChip data processing using subset quantile normalization for accurate DNA methylation estimation. Epigenomics. 2012;4(3):325-41.

22. McConnell R, Berhane K, Yao L, Jerrett M, Lurmann F, Gilliland F, et al. Traffic, susceptibility, and childhood asthma. Environ Health Perspect. 2006;114(5):766-72.

23. Noushmehr H, Weisenberger DJ, Diefes K, Phillips HS, Pujara K, Berman BP, et al. Identification of a CpG island methylator phenotype that defines a distinct subgroup of glioma. Cancer Cell. 2010;17(5):510-22.

24. Aryee MJ, Jaffe AE, Corrada-Bravo H, Ladd-Acosta C, Feinberg AP, Hansen KD, et al. Minfi: a flexible and comprehensive Bioconductor package for the analysis of Infinium DNA methylation microarrays. Bioinformatics. 2014;30(10):1363-9.

25. Triche TJ, Jr., Weisenberger DJ, Van Den Berg D, Laird PW, Siegmund KD. Low-level processing of Illumina Infinium DNA Methylation BeadArrays. Nucleic Acids Res. 2013;41(7):e90.

26. Newschaffer CJ, Croen LA, Fallin MD, Hertz-Picciotto I, Nguyen DV, Lee NL, et al. Infant siblings and the investigation of autism risk factors. J Neurodev Disord. 2012;4(1):7.

27. Leek JT, Storey JD. Capturing heterogeneity in gene expression studies by surrogate variable analysis. PLoS Genet. 2007;3(9):1724-35.

28. Heude B, Forhan A, Slama R, Douhaud L, Bedel S, Saurel-Cubizolles MJ, et al. Cohort Profile: The EDEN mother-child cohort on the prenatal and early postnatal determinants of child health and development. Int J Epidemiol. 2016;45(2):353-63.

29. Schoeters G, Den Hond E, Colles A, Loots I, Morrens B, Keune H, et al. Concept of the Flemish human biomonitoring programme. Int J Hyg Environ Health. 2012;215(2):102-8.

30. Koppen G, Den Hond E, Nelen V, Van De Mieroop E, Bruckers L, Bilau M, et al. Organochlorine and heavy metals in newborns: results from the Flemish Environment and Health Survey (FLEHS 2002-2006). Environ Int. 2009;35(7):1015-22.

31. Maksimovic J, Gordon L, Oshlack A. SWAN: Subset-quantile within array normalization for illumina infinium HumanMethylation450 BeadChips. Genome Biol. 2012;13(6):R44.

32. Houseman EA, Accomando WP, Koestler DC, Christensen BC, Marsit CJ, Nelson HH, et al. DNA methylation arrays as surrogate measures of cell mixture distribution. BMC Bioinformatics. 2012;13:86.

33. Reinius LE, Acevedo N, Joerink M, Pershagen G, Dahlen SE, Greco D, et al. Differential DNA methylation in purified human blood cells: implications for cell lineage and studies on disease susceptibility. PLoS One. 2012;7(7):e41361.

34. Slieker RC, Bos SD, Goeman JJ, Bovee JV, Talens RP, van der Breggen R, et al. Identification and systematic annotation of tissue-specific differentially methylated regions using the Illumina 450k array. Epigenetics Chromatin. 2013;6(1):26.

35. Kumar R, Nguyen EA, Roth LA, Oh SS, Gignoux CR, Huntsman S, et al. Factors associated with degree of atopy in Latino children in a nationwide pediatric sample: the Genes-environments and Admixture in Latino Asthmatics (GALA II) study. J Allergy Clin Immunol. 2013;132(4):896-905 e1.

36. Nishimura KK, Galanter JM, Roth LA, Oh SS, Thakur N, Nguyen EA, et al. Early-life air pollution and asthma risk in minority children. The GALA II and SAGE II studies. Am J Respir Crit Care Med. 2013;188(3):309-18.

37. Oh SS, Tcheurekdjian H, Roth LA, Nguyen EA, Sen S, Galanter JM, et al. Effect of secondhand smoke on asthma control among black and Latino children. J Allergy Clin Immunol. 2012;129(6):1478-83 e7.

38. Dedeurwaerder S, Defrance M, Calonne E, Denis H, Sotiriou C, Fuks F. Evaluation of the Infinium Methylation 450K technology. Epigenomics. 2011;3(6):771-84.

39. L'Abee C, Sauer PJ, Damen M, Rake JP, Cats H, Stolk RP. Cohort Profile: the GECKO Drenthe study, overweight programming during early childhood. Int J Epidemiol. 2008;37(3):486-9.

40. Jaddoe VW, van Duijn CM, Franco OH, van der Heijden AJ, van Iizendoorn MH, de Jongste JC, et al. The Generation R Study: design and cohort update 2012. Eur J Epidemiol. 2012;27(9):739-56.

41. Guillemette L, Allard C, Lacroix M, Patenaude J, Battista MC, Doyon M, et al. Genetics of Glucose regulation in Gestation and Growth (Gen3G): a prospective prebirth cohort of mother-child pairs in Sherbrooke, Canada. BMJ Open. 2016;6(2):e010031.

42. Paternoster L, Evans DM, Nohr EA, Holst C, Gaborieau V, Brennan P, et al. Genome-wide population-based association study of extremely overweight young adults--the GOYA study. PLoS One. 2011;6(9):e24303.

43. van der Valk RJ, Duijts L, Timpson NJ, Salam MT, Standl M, Curtin JA, et al. Fraction of exhaled nitric oxide values in childhood are associated with 17q11.2-q12 and 17q12-q21 variants. J Allergy Clin Immunol. 2014;134(1):46-55.

44. Vilahur N, Bustamante M, Morales E, Motta V, Fernandez MF, Salas LA, et al. Prenatal exposure to mixtures of xenoestrogens and genome-wide DNA methylation in human placenta. Epigenomics. 2016;8(1):43-54.

45. van Iterson M, Tobi EW, Slieker RC, den Hollander W, Luijk R, Slagboom PE, et al. MethylAid: visual and interactive quality control of large Illumina 450k datasets. Bioinformatics. 2014;30(23):3435-7.

46. Lehne B, Drong AW, Loh M, Zhang W, Scott WR, Tan ST, et al. A coherent approach for analysis of the Illumina HumanMethylation450 BeadChip improves data quality and performance in epigenome-wide association studies. Genome Biol. 2015;16:37.

47. Johnson WE, Li C, Rabinovic A. Adjusting batch effects in microarray expression data using empirical Bayes methods. Biostatistics. 2007;8(1):118-27.

48. Busse WW, Mitchell H. Addressing issues of asthma in inner-city children. The Journal of allergy and clinical immunology. 2007;119(1):43-9.

49. Expert Panel Report 3 (EPR-3): Guidelines for the Diagnosis and Management of Asthma-Summary Report 2007. The Journal of allergy and clinical immunology. 2007;120(5 Suppl):S94-138.

50. Aryee MJ, Jaffe AE, Corrada-Bravo H, Ladd-Acosta C, Feinberg AP, Hansen KD, et al. Minfi: a flexible and comprehensive Bioconductor package for the analysis of Infinium DNA methylation microarrays. Bioinformatics. 2014.

51. Leek JT, Scharpf RB, Bravo HC, Simcha D, Langmead B, Johnson WE, et al. Tackling the widespread and critical impact of batch effects in high-throughput data. Nature reviews Genetics. 2010;11(10):733-9.

52. Yang IV, Pedersen BS, Liu A, O'Connor GT, Teach SJ, Kattan M, et al. DNA methylation and childhood asthma in the inner city. The Journal of allergy and clinical immunology. 2015;136(1):69-80.

53. Wang D, Yan L, Hu Q, Sucheston LE, Higgins MJ, Ambrosone CB, et al. IMA: an R package for high-throughput analysis of Illumina's 450K Infinium methylation data. Bioinformatics. 2012;28(5):729-30.

54. Magnus P, Birke C, Vejrup K, Haugan A, Alsaker E, Daltveit AK, et al. Cohort Profile Update: The Norwegian Mother and Child Cohort Study (MoBa). Int J Epidemiol. 2016;45(2):382-8.

55. Ronningen KS, Paltiel L, Meltzer HM, Nordhagen R, Lie KK, Hovengen R, et al. The biobank of the Norwegian Mother and Child Cohort Study: a resource for the next 100 years. Eur J Epidemiol. 2006;21(8):619-25.

56. Magnus P, Irgens LM, Haug K, Nystad W, Skjaerven R, Stoltenberg C, et al. Cohort profile: the Norwegian Mother and Child Cohort Study (MoBa). Int J Epidemiol. 2006;35(5):1146-50.

57. Haberg SE, London SJ, Nafstad P, Nilsen RM, Ueland PM, Vollset SE, et al. Maternal folate levels in pregnancy and asthma in children at age 3 years. J Allergy Clin Immunol. 2011;127(1):262-4, 4 e1.

58. Joubert BR, Haberg SE, Nilsen RM, Wang X, Vollset SE, Murphy SK, et al. 450K epigenome-wide scan identifies differential DNA methylation in newborns related to maternal smoking during pregnancy. Environ Health Perspect. 2012;120(10):1425-31.

59. Joubert BR, Felix JF, Yousefi P, Bakulski KM, Just AC, Breton C, et al. DNA Methylation in Newborns and Maternal Smoking in Pregnancy: Genome-wide Consortium Meta-analysis. Am J Hum Genet. 2016;98(4):680-96.

60. Leek JT, Johnson WE, Parker HS, Jaffe AE, Storey JD. The sva package for removing batch effects and other unwanted variation in high-throughput experiments. Bioinformatics. 2012;28(6):882-3.

61. Wilcox AJ, Lie RT, Solvoll K, Taylor J, McConnaughey DR, Abyholm F, et al. Folic acid supplements and risk of facial clefts: national population based case-control study. BMJ. 2007;334(7591):464.

62. Markunas CA, Xu Z, Harlid S, Wade PA, Lie RT, Taylor JA, et al. Identification of DNA methylation changes in newborns related to maternal smoking during pregnancy. Environ Health Perspect. 2014;122(10):1147-53.

63. Davis S, Du P, Bilke S, Triche TJ, Jr., Bootwalla M. methylumi: Handle Illumina methylation data. 2015.

64. Xu Z, Niu L, Li L, Taylor JA. ENmix: a novel background correction method for Illumina HumanMethylation450 BeadChip. Nucleic Acids Res. 2016;44(3):e20.

65. Bolstad BM, Irizarry RA, Astrand M, Speed TP. A comparison of normalization methods for high density oligonucleotide array data based on variance and bias. Bioinformatics. 2003;19(2):185-93.

66. Niu L, Xu Z, Taylor JA. RCP: a novel probe design bias correction method for Illumina Methylation BeadChip. Bioinformatics. 2016.

67. Hoyo C, Murtha AP, Schildkraut JM, Forman MR, Calingaert B, Demark-Wahnefried W, et al. Folic acid supplementation before and during pregnancy in the Newborn Epigenetics STudy (NEST). BMC Public Health. 2011;11(1):46.

68. Wijga AH, Kerkhof M, Gehring U, de Jongste JC, Postma DS, Aalberse RC, et al. Cohort profile: the prevention and incidence of asthma and mite allergy (PIAMA) birth cohort. Int J Epidemiol. 2014;43(2):527-35.

69. Bousquet J, Anto JM, Akdis M, Auffray C, Keil T, Momas I, et al. Paving the way of systems biology and precision medicine in allergic diseases: The MeDALL success story. Allergy. 2016.

70. Zhang B, Gaiteri C, Bodea LG, Wang Z, McElwee J, Podtelezhnikov AA, et al. Integrated systems approach identifies genetic nodes and networks in late-onset Alzheimer's disease. Cell. 2013;153(3):707-20.

71. Farchi S, Forastiere F, Vecchi Brumatti L, Alviti S, Arnofi A, Bernardini T, et al. Piccolipiu, a multicenter birth cohort in Italy: protocol of the study. BMC Pediatr. 2014;14:36.

72. Girchenko P, Lahti M, Tuovinen S, Savolainen K, Lahti J, Binder EB, et al. Cohort Profile: Prediction and Prevention of Preeclampsia and Intrauterine Growth Restriction (PREDO) Study. Int J Epidemiol.*in press*.

73. Oken E, Baccarelli AA, Gold DR, Kleinman KP, Litonjua AA, De Meo D, et al. Cohort profile: project viva. Int J Epidemiol. 2015;44(1):37-48.

74. Huang RC, Mori TA, Beilin LJ. Early life programming of cardiometabolic disease in the Western Australian pregnancy cohort (Raine) study. Clin Exp Pharmacol Physiol. 2012;39(11):973-8.

75. Newnham JP, Evans SF, Michael CA, Stanley FJ, Landau LI. Effects of frequent ultrasound during pregnancy: a randomised controlled trial. Lancet. 1993;342(8876):887-91.

76. Schendel DE, Diguiseppi C, Croen LA, Fallin MD, Reed PL, Schieve LA, et al. The Study to Explore Early Development (SEED): a multisite epidemiologic study of autism by the Centers for Autism and Developmental Disabilities Research and Epidemiology (CADDRE) network. J Autism Dev Disord. 2012;42(10):2121-40.

77. DiGuiseppi CG, Daniels JL, Fallin DM, Rosenberg SA, Schieve LA, Thomas KC, et al. Demographic profile of families and children in the Study to Explore Early Development (SEED): Case-control study of autism spectrum disorder. Disabil Health J. 2016;9(3):544-51.

78. Almqvist C, Ortqvist AK, Ullemar V, Lundholm C, Lichtenstein P, Magnusson PK. Cohort Profile: Swedish Twin Study on Prediction and Prevention of Asthma (STOPPA). Twin Res Hum Genet. 2015;18(3):273-80.

79. Anckarsater H, Lundstrom S, Kollberg L, Kerekes N, Palm C, Carlstrom E, et al. The Child and Adolescent Twin Study in Sweden (CATSS). Twin Res Hum Genet. 2011;14(6):495-508.

80. Assenov Y, Muller F, Lutsik P, Walter J, Lengauer T, Bock C. Comprehensive analysis of DNA methylation data with RnBeads. Nat Methods. 2014;11(11):1138-40.

81. Zetterqvist J, Sjölander A. Doubly Robust Estimation with the R Package drgee. Epidemiol Methods. 2015;4(1):69-86.
